# The effect of calorie intake, fasting, and dietary composition on metabolic health and gut microbiota in mice

**DOI:** 10.1186/s12915-021-00987-5

**Published:** 2021-03-19

**Authors:** Ziyi Zhang, Xiaoyu Chen, Yuh Jiun Loh, Xin Yang, Chenhong Zhang

**Affiliations:** State Key Laboratory of Microbial Metabolism, School of Life Sciences and Biotechnology, Shanghai Jiao Tong University, Shanghai, 200240 China

**Keywords:** Intermittent fasting, Calorie restriction, High-fat diet, Gut microbiota, *Lactobacillus murinus*

## Abstract

**Background:**

Calorie restriction (CR) and intermittent fasting (IF) can promote metabolic health through a process that is partially mediated by gut microbiota modulation. To compare the effects of CR and IF with different dietary structures on metabolic health and the gut microbiota, we performed an experiment in which mice were subjected to a CR or IF regimen and an additional IF control (IF^Ctrl^) group whose total energy intake was not different from that of the CR group was included. Each regimen was included for normal chow and high-fat diet.

**Results:**

We showed that in normal-chow mice, the IF^Ctrl^ regimen had similar positive effects on glucose and lipid metabolism as the CR regimen, but the IF regimen showed almost no influence compared to the outcomes observed in the ad libitum group. IF also resulted in improvements, but the effects were less marked than those associate with CR and IF^Ctrl^ when the mice were fed a high-fat diet. Moreover, CR created a stable and unique gut microbial community, while the gut microbiota shaped by IF exhibited dynamic changes in fasting-refeeding cycles. At the end of each cycle, the gut microbiota of the IF^Ctrl^ mice was similar to that of the CR mice, and the gut microbiota of the IF mice was similar to that of the ad libitum group. When the abundance of *Lactobacillus murinus* OTU2 was high, the corresponding metabolic phenotype was improved regardless of eating pattern and dietary structure, which might be one of the key bacterial groups in the gut microbiota that is positively correlated with metabolic amelioration.

**Conclusion:**

There are interactions among the amount of food intake, the diet structure, and the fasting time on metabolic health. The structure and composition of gut microbiota modified by dietary regimens might contribute to the beneficial effects on the host metabolism.

**Supplementary Information:**

The online version contains supplementary material available at 10.1186/s12915-021-00987-5.

## Background

Calorie restriction (CR) refers to reducing the daily calorie intake by 15 to 40% without leading to malnutrition. CR has shown numerous beneficial effects on health and metabolism in various model organisms and humans, and these effects include attenuating the inflammatory state, preventing the occurrence of metabolic syndrome and extending the lifespan [1–3]. In a mouse model of CR, a reduction in energy intake can cause food to be consumed within a short period after it is provided, which leads to a longer fasting period until the next supply of food becomes available [4]. Since the aforementioned study on CR was conducted, many researchers have introduced different types of intermittent fasting (IF). In contrast to traditional CR paradigms, IF refers to a variety of eating patterns in which no or few calories are consumed for time periods that range from 12 h to several days on a recurring basis, and these patterns include daily time-restricted feeding, alternate-day fasting and 5:2 intermittent fasting (fasting for 2 days each week) [5–10]. Based on a meta-analysis of previous studies, IF exerts metabolic effects similar to those of CR, such as improving glucose metabolism by lowering insulin resistance [5, 11–14]. To effectively apply CR and IF in clinical practice, a strict comparison of the impacts of these dietary regimens with different dietary compositions is needed.

Although preclinical studies and clinical trials have shown that IF has broad-spectrum benefits for many health conditions, following CR or IF for long periods is a major challenge in the application of fasting-based interventions for the treatment of metabolic syndromes in humans [11, 15]. Further understanding the processes that link IF with broad health benefits might help us develop targeted pharmacologic therapies that mimic the effects of IF without the need to substantially alter an individual’s feeding habits. The mechanisms underlying the metabolism-modifying efficacy of CR and/or IF involve complicated pathways, and the gut microbiota is considered one of the important mediators of interaction between these dietary regimens and host metabolism [16]. It has been established that the total amount of food consumed, the dietary composition, and the eating pattern affect metabolism by regulating the gut microbiome. Previous studies have revealed that both short- and long-term CR significantly change the gut microbiota structure, and only 2 weeks of CR intervention induced the establishment of a *Lactobacillus*-dominant microbial community in the mouse gut, which decreased the levels of circulating microbial antigens and systemic inflammatory markers such as tumor necrosis factor alpha (TNF-α) [17–20]. In obese human adolescents, a 1-year CR period significantly reduces the Firmicutes to Bacteroidetes ratio and enriched beneficial microorganisms such as *Bacteroides*, *Roseburia*, *Faecalibacterium*, and *Clostridium XIVa* [21]. As an alternative to CR, recent studies have shown that IF also exerts a significant effect on the gut microbiota. The results from a study on the prevention of retinopathy in db/db mice showed an increased level of Firmicutes and reduced Verrucomicrobia and Bacteroidetes after IF intervention [22]. Another study revealed that IF increases the gut bacterial richness and altered its composition and related metabolic pathways [23]. Microbiota-depleted mice treated with antibiotics show resistance to the body weight loss and decrease in the blood glucose level induced by CR [24]. Under an every-other-day fasting regimen, white adipose tissue exhibited beigeing in the control group and not in the microbiota-depleted groups [24, 25]. The above-mentioned findings indicate that the gut microbiota is a key intermediary factor. Therefore, the specific similarities and differences in the impact on the gut microbiota between these two dietary regimens with similar health effects need to be further studied.

The gut microbiota responds rapidly to dietary changes. In both rodents and humans, modifying the intake of dietary macronutrients significantly altered the gut microbiome within a single day [26–28]. Our previous research found that the gut microbiota of mice became markedly different from that of the mice in the normal chow group after only 2 days of reduced calorie intake [17]. In the case of the IF regimen, the metabolism of the host is different in the fasting and refeeding stages. Ketogenesis occurs during the fasting period, while glucose level rises after eating resumes, and thus, the energy supply shifts from ketones to glucose [7]. However, there have been a very limited number of studies that have focused on differences in gut microbiota between fasting days and refeeding days in the cycle of IF [23, 25, 29].

To compare the health improvement effects of CR and IF and their effects on the gut microbiota and to identify the specific members in the microbial community that respond to these dietary interventions, we designed experiments in which mice were subjected to the CR or IF regimen with normal chow or a high-fat diet. In humans, the three most widely studied intermittent-fasting regimens are alternate-day fasting, 5:2 intermittent fasting, and daily time-restricted feeding [6, 7]. We then used the classic 5:2 IF regimen, which refers to 2-day fasting followed by 5-day free feeding [30]. Because previous studies have shown that mice consume too much food during the refeeding days in IF, an additional IF control (IF^Ctrl^) group was established to further control the caloric intake in our study. The total energy intake of the IF^Ctrl^ group was not different from that of the CR group. We showed that among mice given normal chow, the IF^Ctrl^ regimen exerted similar positive effects on glucose and lipid metabolism as the CR regimen, but the IF regimen had almost no influence compared with the outcomes observed in the ad libitum group. However, when the mice were fed a high-fat diet, IF also resulted in improvements, but these improvements were less marked than those observed in the CR and IF^Ctrl^ groups. Moreover, CR created a stable and unique gut microbial community in the mice fed the normal chow and those fed the high-fat diet, whereas the gut microbiota shaped by IF exhibited dynamic changes during the fasting-refeeding cycles. At the end of each cycle, the gut microbiota of the IF^Ctrl^ mice was similar to that of the CR mice, and the gut microbiota of the IF mice was similar to that of the ad libitum group. We also identified the members of the gut microbiota that respond to the various dietary regimens, which might be associated with the observed improvements in metabolic phenotypes.

## Results

### The effect of the three dietary regimens on the physiology and metabolism of normal chow-fed mice

To investigate the effect of the three dietary regimens on physiology and metabolism in normal chow-fed mice, 8-week-old male C57BL/6 J mice were randomly assigned to four groups: (1) the control group (NC + AL) received a normal chow diet ad libitum, (2) the calorie-restricted group (NC + CR) was fed 70% of the ad libitum intake every day, (3) the group subjected to the IF regimen (NC + IF) had a 2-day (days 1–2) fasting period followed by a 5-day (days 3–7) ad libitum feeding period weekly, and (4) the group subjected to the IF^Ctrl^ regimen (NC + IF^Ctrl^) had a 2-day fasting period followed by a 5-day daily feeding period in which they consumed the NC + AL daily intake amount (normal amounts of food) weekly to prevent overeating on refeeding days (Additional file 1).

**Fig. 1:**
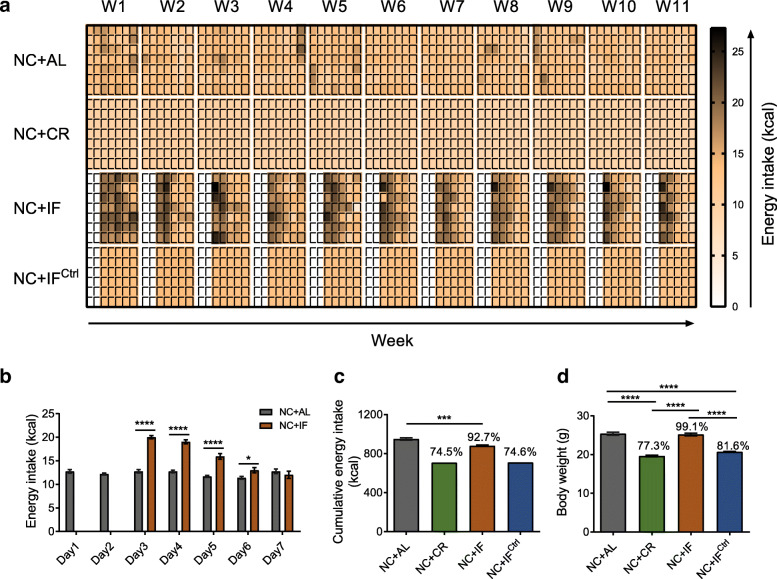
Energy intake and body weight in NC-fed mice. **a** Daily energy intake of NC-fed groups. W, week. **b** Average energy intake of the NC + IF group during the 11-week intervention compared with that of the NC + AL group. **c** Cumulative energy intake and **d** body weights of NC-fed groups after 11 weeks of intervention on day 7 of week 11. Data are presented as the mean ± S.E.M. For each group, *n* = 6–7. Data were analyzed using the unpaired *t* test (two-tailed) in **b** and **c**. Data were analyzed using one-way ANOVA followed by Tukey post hoc test in **d**. *P < 0.05, **P < 0.01, ***P < 0.001, ****P < 0.0001

**Fig. 2:**
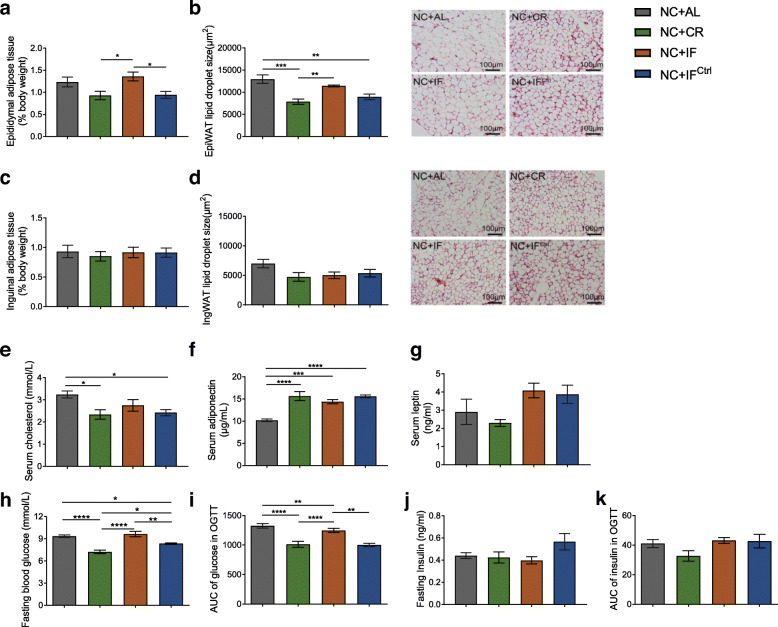
Metabolic parameters of NC-fed mice. **a** Epididymal fat mass as a percentage of body weight in NC-fed mice. **b** Mean lipid droplet area of adipocytes and representative HE-stained histological sections of EpiWAT. **c** Inguinal fat mass as a percentage of body weight in NC-fed mice. **d** Mean lipid droplet area of adipocytes and representative HE-stained histological sections of IngWAT. **e** Serum cholesterol level. **f** Serum adiponectin level. **g** Serum leptin level. **h** Fasting blood glucose. **i** Areas under the curve (AUC) of serum glucose in the oral glucose tolerance test (OGTT). **j** Fasting serum insulin. **k** AUC of serum insulin in the OGTT. Mice were tested after 11 weeks of intervention on day 7 of week 11. Data are presented as the mean ± S.E.M. For each group, *n* = 6–7. Data were analyzed using one-way ANOVA followed by Tukey’s post hoc test. *P < 0.05, **P < 0.01, ***P < 0.001, ****P < 0.0001

On average, in the 11-week trial, compared with the NC + AL group, NC + IF mice consumed dramatically more energy in the first 4 days after refeeding (days 3–6) and ate slightly less on the last refeeding day (day 7; Fig. 1a, b). In all eleven fasting-refeeding cycles, the total energy consumption in NC + IF mice was 92.7% of that in the NC + AL group, and the total energy intakes of the NC + CR group and NC + IF^Ctrl^ group were approximately equivalent, which were approximately 75% of that in NC + AL mice (Fig. 1c). Compared to their ad libitum counterparts, the NC + CR and NC + IF^Ctrl^ mice had significantly lower body weights (Fig. 1d and Additional file 2), decreases in the weight of epididymal white adipose tissue (EpiWAT) and the mean size of lipid droplets in EpiWAT, and lower serum cholesterol levels (Fig. 2a–e and Additional file 3, 4). However, there was no significant difference in body weight, adipose tissue, or serum cholesterol between the NC + IF and NC + AL mice. Furthermore, NC + CR and NC + IF^Ctrl^ mice had significantly reduced fasting blood glucose levels and enhanced glucose tolerance, while the NC + IF group had similar levels of these parameters as the NC + AL mice (Fig. 2h–i and Additional file 5). Unlike the above parameters, the level of serum adiponectin was significantly increased in all the mice from the NC + CR, NC + IF, and NC + IF^Ctrl^ groups compared to the ad libitum control group (Fig. 2f). None of the three dietary interventions based on normal chow affected serum leptin or insulin secretion (Fig. 2g, j and k and Additional file 5). In previous studies, glucose and lipid metabolism in mice with 30% caloric restriction of normal chow was significantly improved compared to that in their ad libitum counterparts [17, 19, 31]. Here, we showed that the NC + IF^Ctrl^ regimen had similar positive effects on glucose and lipid metabolism as NC + CR, but the NC + IF regimen only significantly increased the level of serum adiponectin and enhanced glucose tolerance compared to the outcomes observed in the ad libitum group.

### Dynamic changes in the gut microbiota during the fasting-refeeding cycle

**Fig. 3:**
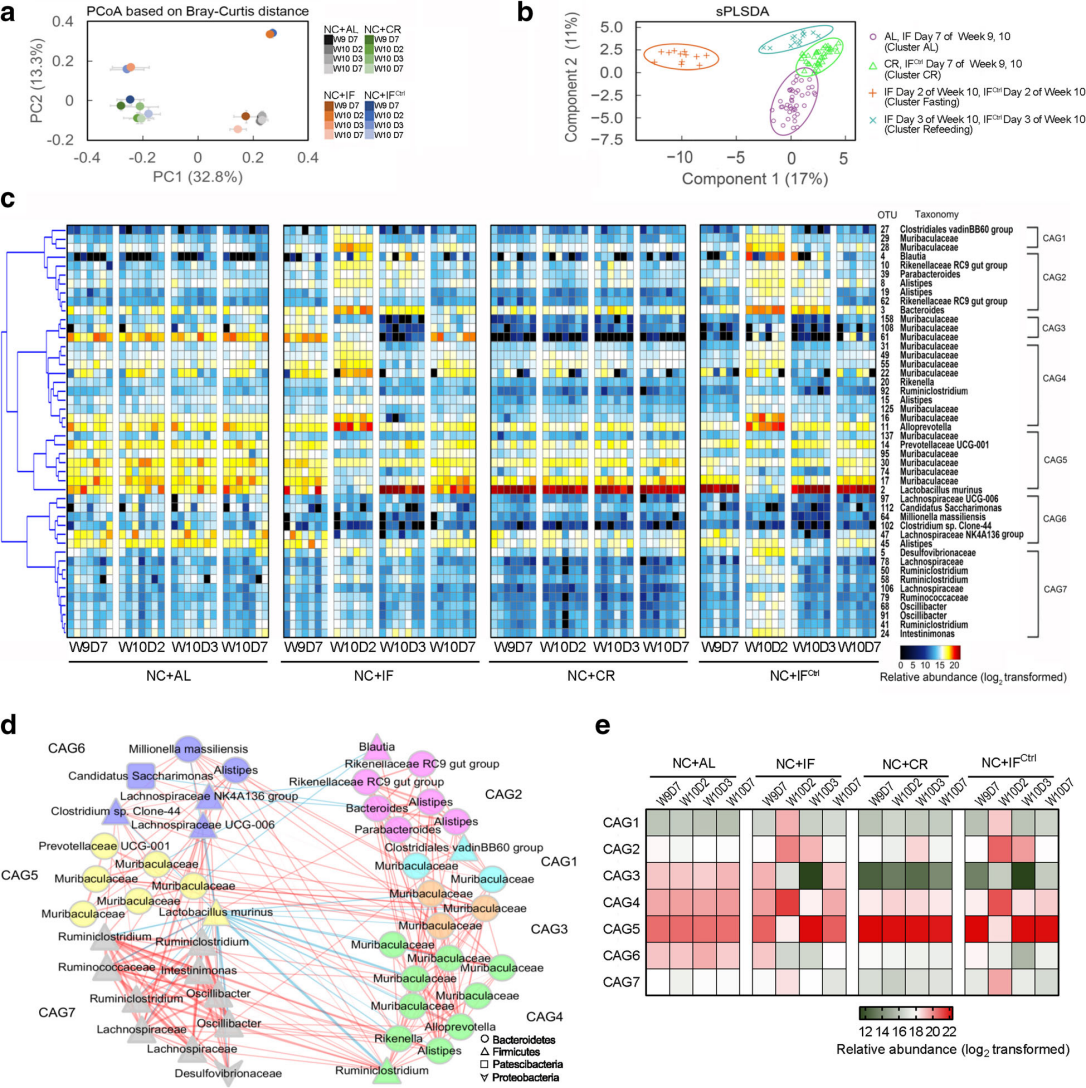
The gut microbiota structural alterations associated with the three dietary intervention regimens in NC-fed mice. **a** Principal coordinate analysis (PCoA) based on the Bray-Curtis distances. **b** Microbiota responding to different interventions based on the sPLS-DA model. All samples from the NC-fed mice at four time points of week 10 were included for discrimination. **c** Heat map of the 46 OTU-level phylotypes identified as key variables for differentiation among the 4 clusters in the gut microbiota of NC-fed mice by sPLS-DA. The color of the spots represents the relative abundance (normalized and log2-transformed) of the OTU in each sample. The OTUs were organized by Spearman’s correlation analysis based on their relative abundances (left side of heat map). OTU ID numbers and the taxa of the OTUs are shown along the right side of the heat map. **d** Coabundance network illustrating the interactions among the 46 key OTUs responding to three dietary intervention regimens in the NC-fed mice. The OTUs were clustered into 7 coabundance groups (CAGs) by PerMANOVA when *P* was < 0.01. Different colors and shapes of nodes represent different CAGs and phyla, respectively. The lines between two nodes represent the correlations between the nodes they connect, with the color saturation and line width indicating the correlation magnitude: red represents a positive correlation, and blue represents a negative correlation. Only lines corresponding to correlations with a magnitude greater than 0.4 were drawn. **e** Heat map of the relative abundance of each CAG on day 7 of week 9 and day 2, day 3, and day 7 of week 10. The color of the spots represents the total relative abundance (normalized and log2-transformed) of all OTUs in each CAG from each group of NC-fed mice. In all graphs, for each group and each time point, *n* = 6–7. W, week; D, day

To determine how the gut microbiota was modulated by these three interventions, fecal samples were collected from all the mice at day 7 of week 9 and day 2, day 3, day 7 of week 10, and the gut microbiota was analyzed through 16S rRNA gene V3-V4 region sequencing. Principal coordinate analysis (PCoA) based on Bray-Curtis distance showed changes in the overall structure of the gut microbiota during the week (Fig. 3a). The gut microbiota in the NC + AL or NC + CR group was relatively stable during the week, and the gut microbiota of the CR group diverged significantly from that of the AL group mainly along the axis of the first principal component (PC1) (*P* < 0.01, permutational multivariate analysis of variance (PerMANOVA) test with 9999 permutations, Additional file 6). At day 7 of week 9 (the beginning of the fasting-refeeding cycle), the structure of the gut microbiota in the NC + IF group was very similar to that in the NC + AL group, while that in the NC + IF^Ctrl^ group was close to that in the NC + CR group. Notably, fasting had a dramatic influence on the gut microbiota in both the NC + IF and NC + IF^Ctrl^ groups. The gut microbiota was not significantly different between these two groups after two days of fasting (day 2) and was much closer to that of the NC + AL group along the axis of PC1 (Fig. 3a and Additional file 6, 7). After 1 day of refeeding (day 3), the gut microbiota greatly changed in these two groups and was similar to that in the NC + CR group. After a 5-day refeeding period (day 7), the gut microbiota in the NC + IF and NC + IF^Ctrl^ groups changed back to that observed at day 7 of the last week.

Based on the PCoA and PerMANOVA test, the gut microbiota of the NC-fed mice could be separated into 4 clusters: (1) the “AL cluster” included all time points of the NC + AL group and day 7 for the two consecutive weeks of the NC + IF group; (2) the “CR cluster” included all time points of the NC + CR group and day 7 for the two consecutive weeks of the NC + IF^Ctrl^ group; (3) the “Fasting cluster” included day 2 of the NC + IF and NC + IF^Ctrl^ groups; (4) the “Refeeding cluster” included day 3 of the NC + IF and NC + IF^Ctrl^ groups, which was confirmed by the Sparse Partial Least Squares Discriminant Analysis (sPLS-DA) model (classification error rate is 0.02, Fig. 3b and Additional file 8). Forty-six operational taxonomic units (OTUs) were identified as features that discriminated the samples among 4 clusters in the sPLS-DA model (Fig. 3c, Additional file 9). Then, we constructed a coabundance network of these 46 key OTUs based on the Spearman correlation coefficients across all groups and time points and clustered them into seven coabundance groups (CAGs) (Fig. 3c–e).

Compared to the NC + AL group, CAG5 was significantly enriched and CAG3, CAG4, CAG6, and CAG7 were significantly reduced in NC + CR mice (Fig. 3c, e, Additional file 10). OTU2 in the genus *Lactobacillus* was the predominant phylotype in CAG5 and showed a strongly negative correlation with the four CAGs that decreased in the CR mice (Fig. 3c–e). Most of the OTUs in CAG3 and CAG4 were in Muribaculaceae and belonged to Bacteroidetes, while most of the OTUs in CAG6 and CAG7 were in Ruminococcaceae and Lachnospiraceae from Firmicutes. These OTU-level alterations induced by CR are consistent with the findings of previous studies [17, 19]. On day 7 of the last week, the characteristics of these bacteria in NC + IF mice were similar to NC + AL mice, while those in NC + IF^Ctrl^ mice were similar to the NC + CR group (Fig. 3c, e). The 2-day fasting changed all of the CAGs in both the NC + IF and NC + IF^Ctrl^ groups, including an increase in CAG1, CAG2, CAG4, and CAG7 and a decrease in CAG3, CAG5, and CAG6 (Fig. 3c, e). In these two fasting groups, CAG2 mainly contained *Bacteroides* OTU3, CAG4 was mainly constructed by *Alloprevotella* OTU11, and Muribaculacea OTU16 became the most abundant bacterial group, while CAG5, containing *Lactobacillus* OTU2, was dramatically reduced to a level significantly lower than that in NC + AL mice. After 1 day of refeeding, most of the seven CAGs decreased, but CAG5 significantly increased in the NC + IF and NC + IF^Ctrl^ groups, even reaching a similar level as that in the NC + CR group (Fig. 3c, e). After a 5-day refeeding period (day 7), the relative abundance of all the CAGs in the NC + IF and NC + IF^Ctrl^ groups changed back to that at day 7 of the last week (Fig. 3c, e).

Considered together, our results showed alterations in the structure and components of the gut microbiota induced by three dietary interventions, suggesting that the gut microbiota in NC + IF and NC + IF^Ctrl^ mice exhibited dynamic cyclical changes. Moreover, at the end of the fasting-refeeding cycle (also the beginning of the next cycle), the characteristics of the gut microbiota was just in parallel with the similar positive effects of NC + CR and NC + IF^Ctrl^ but the rare effect of NC + IF on health-improving.

### The effect of three dietary regimens on physiology and metabolism in mice fed a high-fat diet

To evaluate the effect of various eating regimens on the intake of different dietary components, we performed the same dietary treatments in mice fed a high-fat diet (60% energy from fat). Eight-week-old male C57BL/6 J mice were then subjected to one of four eating regimens (HF + AL, HF + CR, HF + IF or HF + IF^Ctrl^) for 11 weeks.

**Fig. 4:**
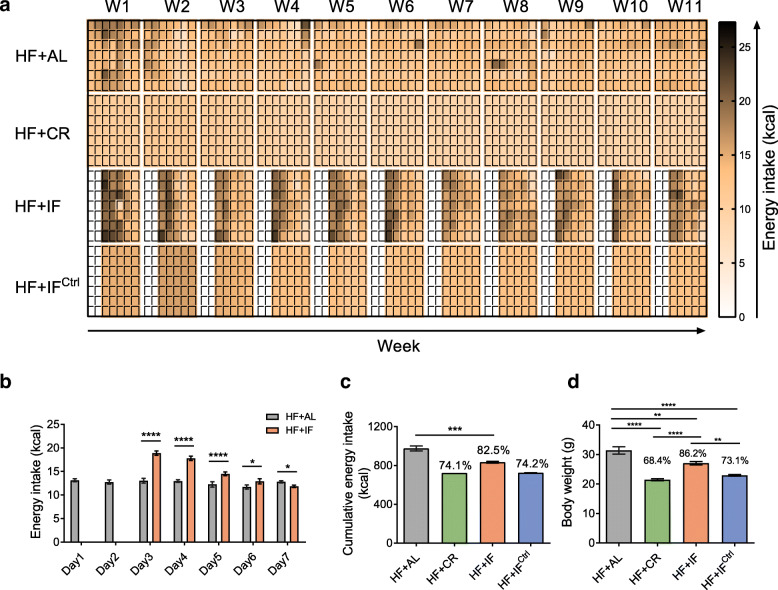
Energy intake and body weight in HF-fed mice. **a** Daily energy intake of HF-fed groups. W, week. **b** Average energy intake of the HF + IF group during the 11-week intervention compared with that of the HF + AL group. **c** Cumulative energy intake and **d** body weight of the HF-fed groups after 11 weeks of intervention on day 7 of week 11. Data are presented as the mean ± S.E.M. For each group, *n* = 6–7. Data were analyzed using the unpaired *t* test (two-tailed) in **b** and **c**. Data were analyzed using one-way ANOVA followed by Tukey post hoc test in **d**. *P < 0.05, **P < 0.01, ***P < 0.001, ****P < 0.0001

**Fig. 5:**
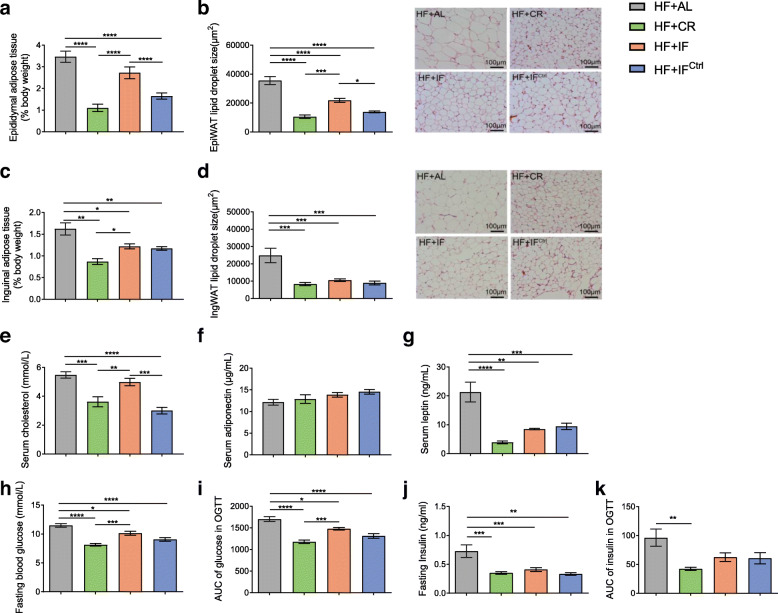
Metabolic parameters of HF-fed mice. **a** Epididymal fat mass as a percentage of body weight in HF-fed mice. **b** Mean lipid droplet area of adipocytes and representative HE-stained histological sections of EpiWAT. **c** Inguinal fat mass as a percentage of body weight in HF-fed mice. **d** Mean lipid droplet area of adipocytes and representative HE-stained histological sections of IngWAT. **e** Serum cholesterol level. **f** Serum adiponectin level. **g** Serum leptin level. **h** Fasting blood glucose. **i** Areas under the curve (AUC) of serum glucose in the oral glucose tolerance test (OGTT). **j** Fasting serum insulin. **k** AUC of serum insulin in the OGTT. Mice were tested after 11 weeks of intervention on day 7 of week 11. Data are presented as the mean ± S.E.M. For each group, *n* = 6–7. Data were analyzed using one-way ANOVA followed by Tukey’s post hoc test. *P < 0.05, **P < 0.01, ***P < 0.001, ****P < 0.0001

The change in food consumption and energy intake in HF + CR, HF + IF or HF + IF^Ctrl^ mice on each day of 1-week fasting-refeeding cycle was similar to that in mice with normal chow compared to their respective ad libitum groups (Fig. 4). Remarkably, the reduction in total calorie intake in HF + IF mice was approximately 17.5%, which was more than twice that in NC + IF mice. At the end of the trial, the body weight of three intervention groups was significantly lower than that in the HF + AL group, while those in the HF + CR and HF + IF^Ctrl^ groups were also significantly lower than that in the HF + IF group (Fig. 4d and Additional file 11). Moreover, all three dietary interventions also attenuated the excessive accumulation of white adipose tissue and the disruption of glucose and lipid metabolism caused by HF feeding, while the effect of HF + IF was less notable than those of the HF + CR and HF + IF^Ctrl^ conditions (Fig. 5 and Additional file 12, 13).

### Three intervention regimens with a high-fat diet altered the gut microbiota

**Fig. 6:**
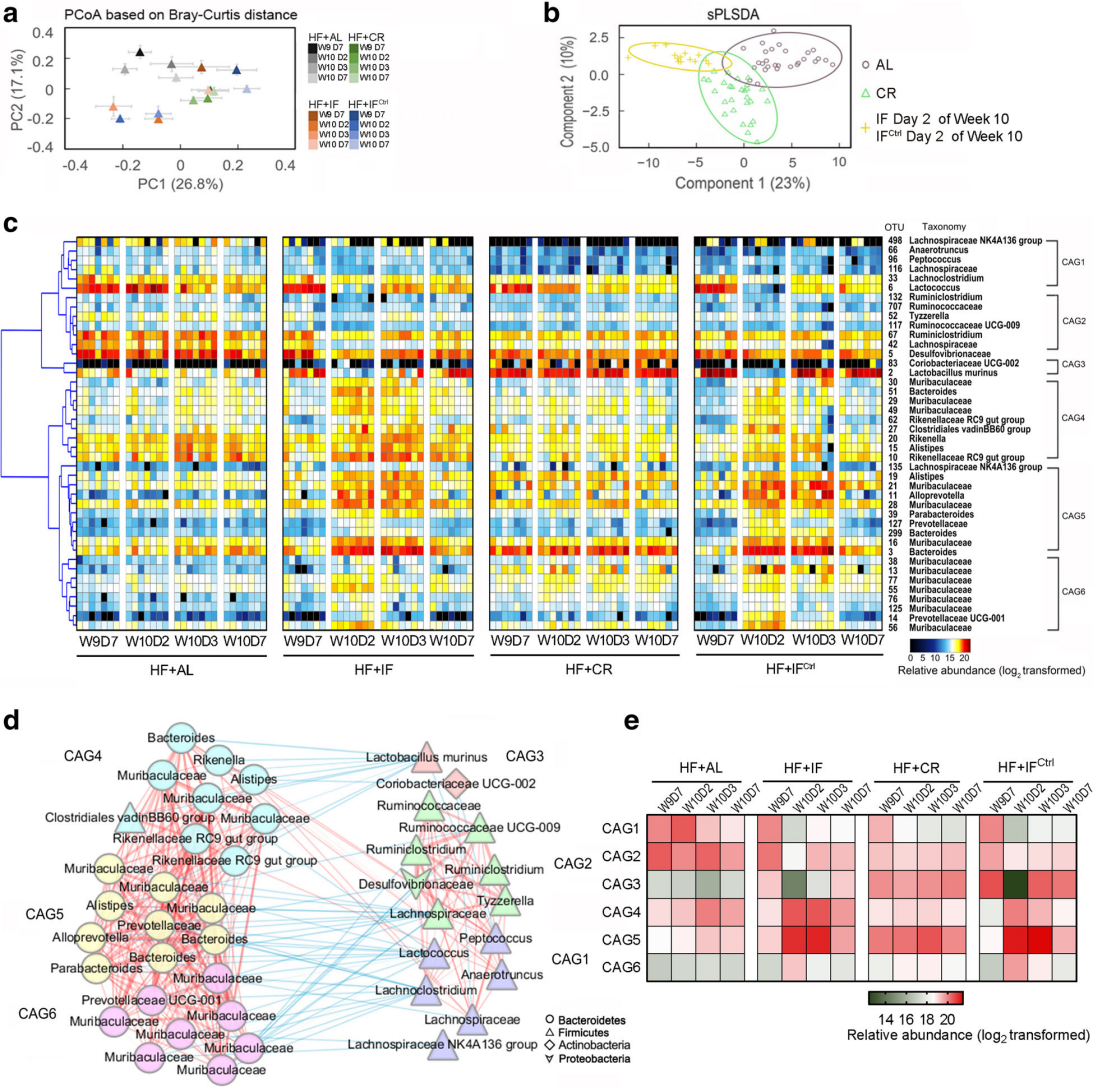
The structure of the gut microbiota altered by the three dietary intervention regimens in HF-fed mice. **a** PCoA based on the Bray-Curtis distances. **b** Microbiota responding to different interventions based on the sPLS-DA model. Samples from the HF + AL and HF + CR groups at four time points and the HF + IF and HF + IF^Ctrl^ groups at day 2 of week 10 were included for discrimination. **c** Heat map of the 42 OTU-level phylotypes identified as key variables for differentiation among the 3 clusters in the gut microbiota of HF-fed mice by sPLS-DA. The color of the spots represents the relative abundance (normalized and log2-transformed) of the OTU in each sample. The OTUs were organized by Spearman’s correlation analysis based on their relative abundances (left side of heat map). OTU ID numbers and the taxa of the OTUs are shown along the right side of the heat map. **d** Coabundance network illustrating the interactions among the 42 key OTUs responding to three dietary intervention regimens in the HF-fed mice. The OTUs were clustered into 6 CAGs by PerMANOVA when *P* was < 0.01. Different colors and shapes of nodes represent different CAGs and phyla, respectively. The lines between two nodes represent the correlations between the nodes they connected, with the color saturation and line width indicating the correlation magnitude: red represents a positive correlation, and blue represents a negative correlation. Only lines corresponding to correlations with a magnitude greater than 0.4 were drawn. **e** Heat map of the relative abundance of each CAG on day 7 of week 9 and day 2, day 3, and day 7 of week 10. The color of the spots represents the total relative abundance (normalized and log2-transformed) of all OTUs in each CAG from each group of HF-fed mice. In all graphs, for each group at each time point, *n* = 6–7. W, week; D, day

The overall structure of the gut microbiota of HF-fed mice at day 7 in week 9 and day 2, day 3, and day 7 in week 10 was also profiled. The PCoA score plot of the Bray-Curtis distance based on OTU data showed that the overall structures of the gut microbiota in the HF + AL and HF + CR groups were relatively stable and that the gut microbiota in the HF + CR group was significantly different from that in the HF + AL group (Fig. 6a and Additional file 14). Moreover, the gut microbiota in HF + IF and HF + IF^Ctrl^ mice exhibited dynamic cyclical changes, but the trajectory was different from that in NC + IF and NC + IF^Ctrl^ mice. The gut microbiota of the HF + IF and HF + IF^Ctrl^ groups at day 7 of week 9 was very similar to that of the HF + CR group and then dramatically changed in the same direction during fasting (day 2). After refeeding, their gut microbiota gradually shifted back to that at day 7 of the last week. The gut microbiota of HF + IF and HF + IF^Ctrl^ mice at all time points was more similar to that of HF + CR mice than that of HF + AL mice (Additional file 15).

Then, we also used sPLS-DA models to identify the features of the gut microbiota in HF + AL and HF + CR mice or HF + IF and HF + IF^Ctrl^ mice on fasting days (classification error rate is 0.01, Fig. 6b and Additional file 16), and 42 OTUs were identified as features of the sPLS-DA model (Fig. 6c, Additional file 17). We next constructed a coabundance network of these 42 key OTUs based on the Spearman correlation coefficients across all groups and time points and clustered them into six coabundance groups (CAGs) (Fig. 6c–e).

Compared with the HF + AL group, the HF + CR group exhibited a decreased abundance of CAG1 and CAG2 and an increased abundance of CAG3, CAG5, and CAG6 (Fig. 6e, Additional file 18). In the fasting period, the abundance of CAG1, CAG2, and CAG3 significantly decreased in the HF + IF and HF + IF^Ctrl^ groups, while CAG4, CAG5, and CAG6 increased. Notably, compared to the gut microbiota in mice fed with normal chow, the OTUs in CAG1 and CAG2 were significantly promoted by a high-fat diet, and treatment with CR, IF, or IF^Ctrl^ could decrease these bacteria. Moreover, consistent with its behavior in mice fed with normal chow, the *Lactobacillus* OTU2 (belonging to CAG3) was the predominant bacterium in the HF + CR, HF + IF, and HF + IF^Ctrl^ groups but was almost eradicated after fasting. The most abundant OTUs in CAG5 were *Bacteroides* OTU3, *Alloprevotella* OTU11, and Muribaculacea OTU16, which were the main members of CAG2 and CAG4 in mice fed with normal chow. Although this CAG in high-fat-fed mice was significantly enriched by fasting, refeeding with a high-fat diet did not reduce its abundance rapidly, indicating that the change tendency of this CAG was different between mice fed with high-fat and normal chow diets.

Overall, these results indicated distinct changes in the gut microbiota in HF-fed mice in response to our three types of interventions. For HF + CR mice, calorie restriction created a stable and unique gut microbial community. For HF + IF and HF + IF^Ctrl^ mice, their gut microbiota had a similar trajectory that changes with the fasting-refeeding cycle.

## Discussion

In the current study, we showed that CR and IF^Ctrl^ had similar positive effects on glucose and lipid metabolism in mice fed normal chow, but compared to the ad libitum group, the IF group only exhibited improvements in blood glucose control and adiponectin levels. In the context of a high-fat diet, IF also resulted in improvements, but these improvements were not as obvious as those in the CR and IF^Ctrl^ groups. CR molded a stable and unique gut microbial community, while the microbiome shaped by IF and IF^Ctrl^ had dynamically periodical changes associated with the fasting-refeeding cycles. Moreover, at the end of the fasting-refeeding cycle, the characteristics of the gut microbiota in CR and IF^Ctrl^ mice were similar, while that in ad libitum and IF mice were same, which were just in parallel with the effects on health-improving.

The current study showed that the amount of food intake, the diet structure, and the fasting time had mutual impacts on glucose and lipid metabolism. Previous studies have found that reducing food intake could notably improve the metabolic status and physiological phenotype in mice, such as increasing glucose-insulin homeostasis and reducing serum levels of proinflammatory factors [17, 19, 32–34]. In a recent study, it was noteworthy that a decrease in energy intake caused food to be consumed in a short time, followed by longer daily fasting periods, implying that the health effects of CR may be partly attributed to prolonged fasting periods [4]. Other mouse experiments also found that IF significantly improved fat loss and insulin sensitivity, accompanied by a reduction in total calorie intake of at least 50% [35, 36]. Among mice fed a high-fat or high-fructose diet, but not a control balanced diet, IF improved glucose and lipid metabolism [35], that is, diet structure may affect the effectiveness of the IF intervention. Since there is an interaction among the effects of food intake, diet structure, and fasting time on metabolic health, it is essential to consider these factors comprehensively in future studies.

There is growing evidence that the quantities of food consumed could regulate gut microbiota [18, 21, 37], and the metabolites produced by the altered microbial community play an important role in promoting nutrient metabolism in the host [38–40]. Implicit in the present findings is that the microbiome shaped by IF and IF^Ctrl^ changed dynamically during the fasting-refeeding cycles and responded very quickly to the dietary changes. Recently, several studies reported the mediating effect of the gut microbiota in the health-promoting effects of IF, such as enrichment of anti-inflammatory related microorganisms, upregulation of short-chain fatty acid production, enhancement of antioxidant microbial metabolic pathways, and increase in ketogenesis in the liver during fasting [23, 41, 42]. However, the above studies did not report the changes in the microbiota during fasting and refeeding periods and did not specify when the samples were collected. In these studies, the postintervention microbiota was used to explain the metabolic changes, but for future studies, it would be meaningful to consider the different profiles of gut bacterial metabolites during fasting and refeeding periods.

Based on the OTU-level analysis of the gut microbiota, we found dynamic changes in the structure of the gut microbiota and identified specific bacterial members whose abundance varies during the fasting-refeeding cycle. In the current work, when the abundance of *Lactobacillus murinus* OTU2 in the gut was high, the corresponding metabolic phenotype at the end of the experimental period was improved, regardless of dietary pattern and structure. Our previous studies showed that a unique *Lactobacillus*-predominated microbial community is attained in mice administered lifelong or short-term CR, and this effect is strongly correlated with an increase in multiple metabolic improvements and a decrease in the levels of circulating microbial antigens and systemic inflammatory markers [17, 18]. Moreover, *Lactobacillus murinus* is more likely to be enriched by CR in a normal rhythm, and simultaneously, the mice exhibited a better metabolic status than those who ate during the day (abnormal rhythm) [19]. We then isolated a *Lactobacillus murinus* strain (named CR147) that represented the most abundant *Lactobacillus* OTU enriched by CR from the feces of mice in the CR group. This *Lactobacillus murinus* CR147 downregulated interleukin-8 production in TNF-α-stimulated Caco-2 cells and significantly increased the lifespan and the brood size of the nematode *Caenorhabditis elegans*. In gnotobiotic mice colonized with the gut microbiota from old mice, this strain decreased the intestinal permeability and serum endotoxin load, which consequently attenuated the inflammation induced by the old microbiota. Data obtained using various experimental systems showed that the *L. murinus* strain isolated from the feces of mice in the CR group was one of the key members contributing to the protection of the gut barrier and the attenuation of chronic systemic inflammation. The representative sequence of OTU2 showed 100% similarity to the V3-V4 regions of the 16S rRNA gene sequence of *L. murinus* CR147, which suggested that OTU2 might be a key bacterium in the gut microbiota and that it vitally contributes to metabolic amelioration. If a core bacterium such as *L. murinus* can be identified in the human gut, it might have the potential to improve metabolism and be a target for metabolic intervention.

## Conclusions

Due to the terrible compliance of humans with respect to interventions regarding diet and feeding habits, their application is a very complicated issue, and there is no simple dietary regimen protocol that is recommended with respect to health, metabolism and weight loss, particularly based on the current animal studies. The importance of our work is to highlight that the amount of food intake, the diet structure, and the fasting time should be integrated when evaluating the effect of CR and IF on human health, when attempting to understand the mechanisms of CR and IF, or when elucidating the relationship between dietary intervention and the gut microbiota. Moreover, identification of the key bacterial group in the gut microbiota affected by the three regimens and positively correlated with metabolic amelioration, such as *L. murinus*, might help us to develop targeted therapies to prevent and treat obesity and metabolic diseases and further solve global public health problems such as metabolic syndrome.

## Methods

### Animal trial and samples

Specific-pathogen-free, 7-week-old C57BL/6 male mice (*n* = 55) were purchased from SLAC Inc. (Shanghai, China). All mice were housed individually and maintained under a 12-h light/dark cycle (lights on at 7:00 AM and off at 7:00 PM) at a temperature of 22 °C ± 3 °C. All animal experimental procedures were approved by Institutional Animal Care and Use Committee of Shanghai Jiao Tong University (No. 2017013). Mice were randomly separated into two groups and subjected to either a normal chow diet (12% energy from fat, Mice maintain diet, ShukeBeita, China) or a high-fat diet (60% energy from fat, D12492, Research Diets, USA) prior to the initiation of the experiments. After 1 week of acclimatization, within each diet group (NC or HF), mice were randomly allocated into one of the following four intervention groups: (1) fed ad libitum (NC + AL or HF + AL), (2) fed with 30% calorie restriction (70% of the ad libitum intake daily) (NC + CR or HF + CR), (3) subjected to the 5:2 IF regimen, which was 2-day (days 1–2) fasting followed by a 5-day (days 3–7) ad libitum period (NC + IF or HF + IF), or (4) subjected to the 5:2 IF^Ctrl^ regimen that was 2-day (days 1–2) fasting followed by a 5-day (days 3–7) feeding period with the average daily intake of the AL group (NC + IF^Ctrl^ or HF + IF^Ctrl^). Food consumption and body weight were measured every day, and food allotments for the CR and IF^Ctrl^ groups were adjusted accordingly. Each group had 6–7 individually caged mice, and the intervention lasted for 11 weeks. We weighed and recorded daily consumption of food between 6:00 PM and 7:00 PM each day, as well as the provided and refused food except in the AL groups. Fresh feces were collected at day 7 of week 9 and day 2, day 3, and day 7 of week 10. All fecal samples were stored at − 80 °C until analysis. At day 7 of week 11, mice were humanely euthanized after 6 h of food deprivation, and epididymal white adipose tissue (EpiWAT), inguinal white adipose tissue (IngWAT), and vastus lateralis muscles were collected and weighed. Blood samples were collected from the orbital vascular plexus, and serum samples were isolated by centrifugation at 3000*g* at 4 °C for 15 min and stored at − 80 °C.

### Oral glucose tolerance test (OGTT)

The OGTT was conducted on day 7 of week 10. After 6 h of food deprivation, glucose was administered to the mice by oral gavage at a dose of 2.0 g/kg of body weight. Blood glucose levels were determined in samples taken from the tip of the tail vein before and 15, 30, 60, 90, and 120 min after glucose administration using a glucometer (ACCUCHEK® Performa, Roche, USA). Blood samples collected before and 15 and 60 min after glucose administration were collected, and serum was isolated by centrifugation at 3000 g at 4 °C for 15 min and stored at − 80 °C.

### H&E staining of white fat tissue and histopathologic analysis

Fresh inguinal fat pads and epididymal fat pads were fixed in 4% paraformaldehyde for 48 h and dehydrated through a series of graded ethanol baths to displace the water before being embedded in paraffin. Samples were sectioned at 5 μm and stained by hematoxylin and eosin (H&E). Digital images of H&E-stained sections were acquired using a Leica DMRBE microscope (Leica Microsystems GmbH, Germany). Adipocyte lipid droplet size (cross-sectional area) was counted by Image Pro Plus 6.0. Droplet areas were determined in at least three histologic sections and 300 total adipocytes for each mouse.

### Serum parameter measurements

Enzyme-linked immunosorbent assay (ELISA) kits were used to determine the amount of serum fasting insulin (10-1249-01; Mercodia, Sweden), leptin (MOB00, R&D Systems, Minneapolis, MN, USA), and adiponectin (MHSTA50; R&D Systems, Minneapolis, MN, USA). All the ELISA kits used in the current study were highly sensitive kits. All operations were performed in accordance with the instructions of the manufacturer.

Serum cholesterol concentrations were detected using the total cholesterol assay kit (A111-1, Nanjing Jiancheng Bioengineering Institute, China) according to the instructions of the manufacturer.

### Fecal DNA extraction and 16S rRNA gene V3-V4 region sequencing

Total microbial DNA from fecal samples collected at day 7 of week 9 and day 2, day 3, and day 7 of week 10 after treatment was extracted, as described previously [43]. According to the manufacturer’s instructions (Part # 15044223Rev. B, Illumina Inc., USA) with improvements as previously described [44], a sequencing library of the 16S rRNA gene V3-V4 region in DNA samples was constructed and sequenced on the Illumina MiSeq platform (Illumina, Inc., USA) using MiSeq reagent kit v3 (600 cycles, catalog no. MS-102-3033; Illumina).

### Analysis of 16S rRNA V3-V4 sequencing data

Unique sequences obtained from high-quality sequencing alignments were divided into operational taxonomic units (OTUs) using the UPARSE algorithm with 97% similarity [45]. The OTU table was fulfilled by dividing all high-quality sequences into their corresponding OTUs at a 97% similarity cutoff with the USEARCH algorithm [46]. A phylogenetic tree was constructed from representative sequences of all OTUs with FastTree [47]. Each OTU representative sequence was identified based on the SILVA rRNA database project (Silva 132).

The sequences of all samples were downsized to 10,000 reads (1000 permutations) to normalize the depth of sequencing. Two samples with fewer than 10,000 high-quality reads were excluded. Further analysis of the microbiota was performed on the QIIME platform (Quantitative Insight Into Microbial Ecology, v1.8.0) [48]. The richness and diversity of each sample were calculated with observed OTUs, Shannon index and Faith’s phylogenetic diversity (PD Whole tree). The whole structural changes of the gut microbiota in the 10th week after intervention were shown by principal coordinate analysis (PCoA) based on the Bray-Curtis distance of the OTU level. Permutational multivariate analysis of variance (PerMANOVA) was applied to test the significance of the differences in the gut microbiota between two groups (9999 permutations).

Sparse partial least squares-discriminant analysis (sPLS-DA) was used to identify the key OTUs that respond to different dietary interventions. Centered log ratio (CLR) transformations were implemented in sPLS-DA to circumvent spurious results. The optimal classification performance of the sPLS-DA model was assessed with the perf function using 5-fold cross-validation repeated 100 times with the smallest error rate. The above statistical analysis was performed using the mixOmics v6.3.1 R package.

The correlations among OTUs were calculated by the Spearman algorithm. PerMANOVA (9999 permutations, *P* < 0.05) based on Spearman correlation coefficients was used to cluster the OTUs into coabundance groups (CAGs) using the R program.

### Statistical analysis

Statistical analysis was carried out using GraphPad Prism version 7 (GraphPad Software, Inc.). One-way analysis of variance (ANOVA), followed by a Tukey’s post hoc test, was used to determine the statistical significance of the physiological data (Figs. 1, 2, 4, 5, Additional file 2 3, 4, 5, 11, 12, 13). The method to analyze variations between two group was selected according to data distribution: unpaired *t* test (two-tailed) was used for those that obey the normal distribution (Figs. 1, 4), otherwise Mann-Whitney *U* test was used (Additional files 7, 9, 10, 15, 17, 18). Differences were considered statistically significant when the *P* value was < 0.05.

## Supplementary Information

**Additional file 1.** Schematic illustration of the 5:2 IF and 5:2 IF^Ctrl^ regimens.

**Additional file 2. **Body weight of NC-fed mice under different intervention regimens. (A) Body weight curves of NC-fed groups. (B) Body weight change curves of NC-fed groups. (C) Body weight changes of NC-fed groups after 11 weeks of intervention on Day 7 of Week 11. Data are presented as the mean ± S.E.M. For each group, *n* = 6–7. Data were analyzed using one-way ANOVA followed by Tukey’s post hoc test. *P < 0.05, **P < 0.01, ***P < 0.001, ****P < 0.0001.

**Additional file 3. **Lipid droplet size profiling of adipocytes of NC-fed mice under three intervention regimens. Lipid droplet size profiling of adipocytes from (A) EpiWAT and (B) IngWAT of NC-fed mice. Mice were tested after 11 weeks of intervention on Day 7 of Week 11. Data are presented as the mean ± S.E.M. For each group, *n* = 6–7. Data were analyzed using one-way ANOVA followed by Dunnett’s multiple comparisons to compare groups with the NC + AL group. *P < 0.05, **P < 0.01, ***P < 0.001, ****P < 0.0001.

**Additional file 4. **Vastus lateralis tissue weights as a percentage of body weight of NC-fed groups. Mice were tested after 11 weeks of intervention on Day 7 of Week 11. Data are presented as the mean ± S.E.M. For each group, *n* = 6–7. Data were analyzed using one-way ANOVA followed by Tukey’s post hoc test.

**Additional file 5. **Glucose metabolism parameters of NC-fed mice. (A) Homeostatic model assessment for insulin resistance (HOMA-IR). (B) Blood glucose curves during the oral glucose tolerance test (OGTT) of NC-fed groups. (C) Serum insulin during the OGTT (0–60 min). Mice were tested after 11 weeks of intervention on Day 7 of Week 11. Data are presented as the mean ± S.E.M. For each group, *n* = 6–7. Data were analyzed using one-way ANOVA followed by Dunnett’s multiple comparisons to compare with the NC + AL group. *P < 0.05, **P < 0.01, ***P < 0.001, ****P < 0.0001.

**Additional file 6. **Bray-Curtis distances of gut microbiota between NC-fed groups at the all time points. Permutational multivariate analysis of variance (PerMANOVA, 9999 permutations) was used to sequentially determine whether the two groups/time points were significantly different. *P < 0.05, **P < 0.01 (with FDR adjustment).

**Additional file 7. **Intraindividual variations in the gut microbiota of Cluster Fasting and Cluster Refeeding compared with Cluster AL and Cluster CR. Mean values ± SEM are shown. Data were analyzed using the Mann-Whitney *U* test. *P < 0.05, **P < 0.01, ***P < 0.001, ****P < 0.0001.

**Additional file 8.** The overall and balanced error rate (BER) of classification in the sPLS-DA model of NC-fed groups.

**Additional file 9.** The taxonomy, co-abundance group and dynamics of the 46 key OTUs in normal-chow mice.

**Additional file 10.** The dynamics of the 7 co-abundance groups in normal-chow mice.

**Additional file 11. **Body weights in HF-fed mice under different intervention regimens. (A) Body weight curves of HF-fed groups. (B) Body weight change curves of HF-fed groups. (C) Body weight changes of HF-fed groups after 11 weeks of intervention on Day 7 of Week 11. Data are presented as the mean ± S.E.M. For each group, *n* = 6–7. Data were analyzed using one-way ANOVA followed by Tukey’s post hoc test. *P < 0.05, **P < 0.01, ***P < 0.001, ****P < 0.0001.

**Additional file 12. **Lipid droplet size profiling of adipocytes of HF-fed mice under three intervention regimens. Lipid droplet size profiling of adipocytes from (A) EpiWAT and (B) IngWAT of HF-fed mice. Mice were tested after 11 weeks of intervention on Day 7 of Week 11. Data are presented as the mean ± S.E.M. For each group, *n* = 6–7. Data were analyzed using one-way ANOVA followed by Dunnett’s multiple comparisons to compare with the HF + AL group. *P < 0.05, **P < 0.01, ***P < 0.001, ****P < 0.0001.

**Additional file 13. **Vastus lateralis tissue weights as a percentage of body weight of HF-fed groups. Mice were tested after 11 weeks of intervention on Day 7 of Week 11. Data are presented as the mean ± S.E.M. For each group, *n* = 6–7. Data were analyzed using one-way ANOVA followed by Tukey’s post hoc test.

**Additional file 14. **Bray-Curtis distances of gut microbiota between HF-fed groups at all time points. Permutational multivariate analysis of variance (PerMANOVA, 9999 permutations) was used to sequentially determine whether the two groups/time points were significantly different. *P < 0.05, **P < 0.01 (with FDR adjustment).

**Additional file 15. **Intraindividual variations in the gut microbiota of Cluster Day2 (IF Day2/IF^Ctrl^ Day2), IF Day3/IF^Ctrl^ Day3, IF Day7 and IF^Ctrl^ Day7 for the two consecutive weeks compared with Cluster AL and Cluster CR. Mean values ± SEMs are shown. Data were analyzed using the Mann-Whitney *U* test. *P < 0.05, **P < 0.01, ***P < 0.001, ****P < 0.0001.

**Additional file 16.** The overall and balanced error rate (BER) of classification in the sPLS-DA model of HF-fed groups.

**Additional file 17.** The taxonomy, co-abundance group and dynamics of the 42 key OTUs in high-fat-diet mice.

**Additional file 18.** The dynamics of the 6 co-abundance groups in high-fat-diet mice.

## Data Availability

Raw Illumina sequence data of the 16S rRNA gene generated in this study are available in the NCBI Sequence Read Archive (SRA) under accession number SRP273232 (https://trace.ncbi.nlm.nih.gov/Traces/sra/?study=SRP273232).

## References

[1] Fontana L, Partridge L (2015). Promoting health and longevity through diet: from model organisms to humans. Cell.

[2] Fontana L, Klein S (2007). Aging, adiposity, and calorie restriction. JAMA.

[3] Heilbronn LK, de Jonge L, Frisard MI, DeLany JP, Larson-Meyer DE, Rood J, Nguyen T, Martin CK, Volaufova J, Most MM (2006). Effect of 6-month calorie restriction on biomarkers of longevity, metabolic adaptation, and oxidative stress in overweight individuals: a randomized controlled trial. JAMA.

[4] Acosta-Rodríguez VA, de Groot MHM, Rijo-Ferreira F, Green CB, Takahashi JS. Mice under caloric restriction self-impose a temporal restriction of food intake as revealed by an automated feeder system. Cell Metab. 2017;26(1):267–77 e2. 10.1016/j.cmet.2017.06.007 PMC5576447 28683292

[5] Di Francesco A, Di Germanio C, Bernier M, de Cabo R (2018). A time to fast. Science.

[6] Anton SD, Moehl K, Donahoo WT, Marosi K, Lee SA, Mainous AG, Leeuwenburgh C, Mattson MP (2018). Flipping the metabolic switch: understanding and applying the health benefits of fasting. Obesity (Silver Spring).

[7] de Cabo R, Mattson MP (2019). Effects of intermittent fasting on health, aging, and disease. N Engl J Med.

[8] Malinowski B, Zalewska K, Węsierska A, Sokołowska MM, Socha M, Liczner G, Pawlak-Osińska K, Wiciński M (2019). Intermittent fasting in cardiovascular disorders-an overview. Nutrients.

[9] Stockman M-C, Thomas D, Burke J, Apovian CM (2018). Intermittent fasting: is the wait worth the weight?. Curr Obes Rep.

[10] Dong TA, Sandesara PB, Dhindsa DS, Mehta A, Arneson LC, Dollar AL, Taub PR, Sperling LS (2020). Intermittent fasting: a heart healthy dietary pattern?. Am J Med.

[11] Harvie MN, Pegington M, Mattson MP, Frystyk J, Dillon B, Evans G, Cuzick J, Jebb SA, Martin B, Cutler RG (2011). The effects of intermittent or continuous energy restriction on weight loss and metabolic disease risk markers: a randomized trial in young overweight women. Int J Obes.

[12] Barnosky AR, Hoddy KK, Unterman TG, Varady KA (2014). Intermittent fasting vs daily calorie restriction for type 2 diabetes prevention: a review of human findings. Transl Res.

[13] Sundfor TM, Svendsen M, Tonstad S (2018). Effect of intermittent versus continuous energy restriction on weight loss, maintenance and cardiometabolic risk: a randomized 1-year trial. Nutr Metab Cardiovasc Dis.

[14] Carter S, Clifton PM, Keogh JB (2016). The effects of intermittent compared to continuous energy restriction on glycaemic control in type 2 diabetes; a pragmatic pilot trial. Diabetes Res Clin Pract.

[15] Harvie M, Wright C, Pegington M, McMullan D, Mitchell E, Martin B, Cutler RG, Evans G, Whiteside S, Maudsley S (2013). The effect of intermittent energy and carbohydrate restriction v. daily energy restriction on weight loss and metabolic disease risk markers in overweight women. Br J Nutr.

[16] Tremaroli V, Bäckhed F (2012). Functional interactions between the gut microbiota and host metabolism. Nature.

[17] Pan F, Zhang L, Li M, Hu Y, Zeng B, Yuan H, Zhao L, Zhang C (2018). Predominant gut Lactobacillus murinus strain mediates anti-inflammaging effects in calorie-restricted mice. Microbiome.

[18] Zhang C, Li S, Yang L, Huang P, Li W, Wang S, Zhao G, Zhang M, Pang X, Yan Z, *et al*. Structural modulation of gut microbiota in life-long calorie-restricted mice. Nat Commun. 2013;4(1):2163. 10.1038/ncomms3163 PMC3717500 23860099

[19] Zhang L, Xue X, Zhai R, Yang X, Li H, Zhao L, Zhang C. Timing of calorie restriction in mice impacts host metabolic phenotype with correlative changes in gut microbiota. mSystems. 2019;4(6):e00348–19. 10.1128/mSystems.00348-19 PMC6890928 31796564

[20] Liu T, Wu Y, Wang L, Pang X, Zhao L, Yuan H, Zhang C. A more robust gut microbiota in calorie-restricted mice is associated with attenuated intestinal injury caused by the chemotherapy drug cyclophosphamide. mBio. 2019;10(2):e02903–18. 10.1128/mBio.02903-18 PMC6414708 30862756

[21] Ruiz A, Cerdó T, Jáuregui R, Pieper DH, Marcos A, Clemente A, García F, Margolles A, Ferrer M, Campoy C (2017). One-year calorie restriction impacts gut microbial composition but not its metabolic performance in obese adolescents. Environ Microbiol.

[22] Beli E, Yan Y, Moldovan L, Vieira CP, Gao R, Duan Y, Prasad R, Bhatwadekar A, White FA, Townsend SD (2018). Restructuring of the gut microbiome by intermittent fasting prevents retinopathy and prolongs survival in db/db mice. Diabetes.

[23] Cignarella F, Cantoni C, Ghezzi L, Salter A, Dorsett Y, Chen L, Phillips D, Weinstock GM, Fontana L, Cross AH, *et al.* Intermittent fasting confers protection in CNS autoimmunity by altering the gut microbiota. Cell Metab. 2018;27(6):1222–35 e6. 10.1016/j.cmet.2018.05.006 PMC6460288 29874567

[24] Wang S, Huang M, You X, Zhao J, Chen L, Wang L, Luo Y, Chen Y (2018). Gut microbiota mediates the anti-obesity effect of calorie restriction in mice. Sci Rep.

[25] Li G, Xie C, Lu S, Nichols RG, Tian Y, Li L, Patel D, Ma Y, Brocker CN, Yan T *et al*: Intermittent fasting promotes white adipose browning and decreases obesity by shaping the gut microbiota. Cell Metab. 2017;26(4):672–85 e4. 10.1016/j.cmet.2017.08.019 PMC5668683 28918936

[26] Faith JJ, McNulty NP, Rey FE, Gordon JI (2011). Predicting a human gut microbiota's response to diet in gnotobiotic mice. Sci (New York).

[27] Turnbaugh PJ, Ridaura VK, Faith JJ, Rey FE, Knight R, Gordon JI (2009). The effect of diet on the human gut microbiome: a metagenomic analysis in humanized gnotobiotic mice. Sci Transl Med.

[28] David LA, Maurice CF, Carmody RN, Gootenberg DB, Button JE, Wolfe BE, Ling AV, Devlin AS, Varma Y, Fischbach MA (2014). Diet rapidly and reproducibly alters the human gut microbiome. Nature.

[29] Zarrinpar A, Chaix A, Yooseph S, Panda S (2014). Diet and feeding pattern affect the diurnal dynamics of the gut microbiome. Cell Metab.

[30] Longo VD, Mattson MP (2014). Fasting: molecular mechanisms and clinical applications. Cell Metab.

[31] Fabbiano S, Suarez-Zamorano N, Chevalier C, Lazarevic V, Kieser S, Rigo D, Leo S, Veyrat-Durebex C, Gaia N, Maresca M (2018). Functional gut microbiota remodeling contributes to the caloric restriction-induced metabolic improvements. Cell Metab.

[32] Speakman JR, Mitchell SE (2011). Caloric restriction. Mol Asp Med.

[33] Mitchell SE, Tang Z, Kerbois C, Delville C, Konstantopedos P, Bruel A, Derous D, Green C, Aspden RM, Goodyear SR (2015). The effects of graded levels of calorie restriction: I. impact of short term calorie and protein restriction on body composition in the C57BL/6 mouse. Oncotarget.

[34] Mitchell SE, Delville C, Konstantopedos P, Hurst J, Derous D, Green C, Chen L, Han JJD, Wang Y, Promislow DEL (2015). The effects of graded levels of calorie restriction: II. Impact of short term calorie and protein restriction on circulating hormone levels, glucose homeostasis and oxidative stress in male C57BL/6 mice. Oncotarget.

[35] Spezani R, da Silva RR, Martins FF, de Souza MT, Aguila MB, Mandarim-de-Lacerda CA (2020). Intermittent fasting, adipokines, insulin sensitivity, and hypothalamic neuropeptides in a dietary overload with high-fat or high-fructose diet in mice. J Nutr Biochem.

[36] Marinho TS, Ornellas F, Barbosa-da-Silva S, Mandarim-de-Lacerda CA, Aguila MB (2019). Beneficial effects of intermittent fasting on steatosis and inflammation of the liver in mice fed a high-fat or a high-fructose diet. Nutrition.

[37] Santacruz A, Marcos A, Wärnberg J, Martí A, Martin-Matillas M, Campoy C, Moreno LA, Veiga O, Redondo-Figuero C, Garagorri JM (2009). Interplay between weight loss and gut microbiota composition in overweight adolescents. Obesity (Silver Spring).

[38] Jandhyala SM, Talukdar R, Subramanyam C, Vuyyuru H, Sasikala M, Nageshwar Reddy D (2015). Role of the normal gut microbiota. World J Gastroenterol.

[39] Gentile CL, Weir TL (2018). The gut microbiota at the intersection of diet and human health. Science (New York).

[40] Xie G, Zhang S, Zheng X, Jia W (2013). Metabolomics approaches for characterizing metabolic interactions between host and its commensal microbes. Electrophoresis.

[41] Zhang X, Zou Q, Zhao B, Zhang J, Zhao W, Li Y, Liu R, Liu X, Liu Z (2020). Effects of alternate-day fasting, time-restricted fasting and intermittent energy restriction DSS-induced on colitis and behavioral disorders. Redox Biol.

[42] Crawford PA, Crowley JR, Sambandam N, Muegge BD, Costello EK, Hamady M, Knight R, Gordon JI (2009). Regulation of myocardial ketone body metabolism by the gut microbiota during nutrient deprivation. Proc Natl Acad Sci U S A.

[43] Godon JJ, Zumstein E, Dabert P, Habouzit F, Moletta R (1997). Molecular microbial diversity of an anaerobic digestor as determined by small-subunit rDNA sequence analysis. Appl Environ Microbiol.

[44] Zhang Q, Wu Y, Wang J, Wu G, Long W, Xue Z, Wang L, Zhang X, Pang X, Zhao Y (2016). Accelerated dysbiosis of gut microbiota during aggravation of DSS-induced colitis by a butyrate-producing bacterium. Sci Rep.

[45] Edgar RC (2013). UPARSE: highly accurate OTU sequences from microbial amplicon reads. Nat Methods.

[46] Edgar RC (2010). Search and clustering orders of magnitude faster than BLAST. Bioinformatics.

[47] Price MN, Dehal PS, Arkin AP (2009). FastTree: computing large minimum evolution trees with profiles instead of a distance matrix. Mol Biol Evol.

[48] Caporaso JG, Kuczynski J, Stombaugh J, Bittinger K, Bushman FD, Costello EK, Fierer N, Pena AG, Goodrich JK, Gordon JI (2010). QIIME allows analysis of high-throughput community sequencing data. Nat Methods.

