# Supplementary figures and images for: The effect of calorie intake, fasting, and dietary composition on metabolic health and gut microbiota in mice
Source: BMC Biol. 2021 Mar 19;19:51. doi: 10.1186/s12915-021-00987-5 (PMC7977615; doi:10.1186/s12915-021-00987-5)

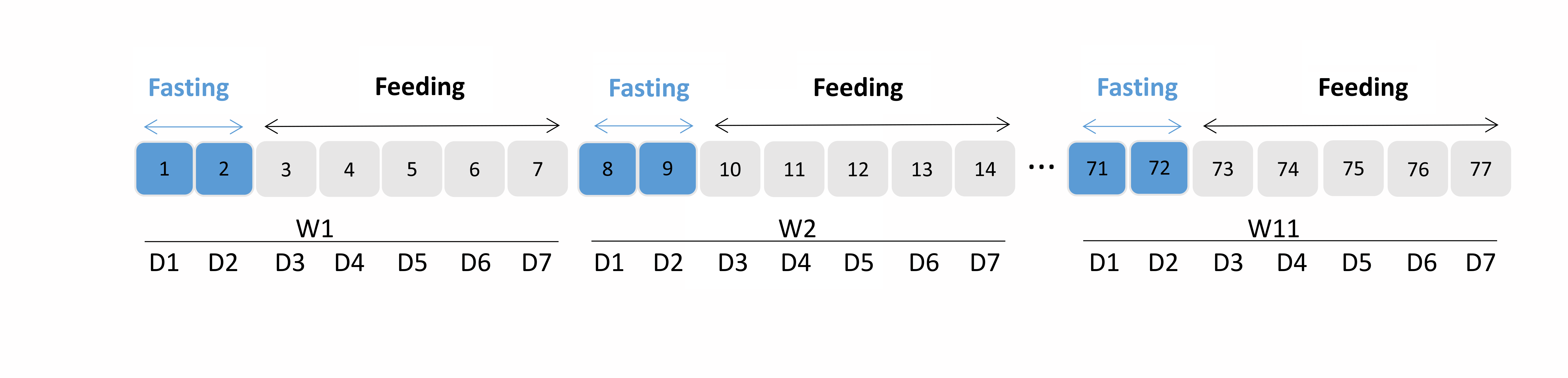

Supplement: Supplementary file 1 — Additional file 1. Schematic illustration of the 5:2 IF and 5:2 IFCtrl regimens. [file 12915_2021_987_MOESM1_ESM.tif]

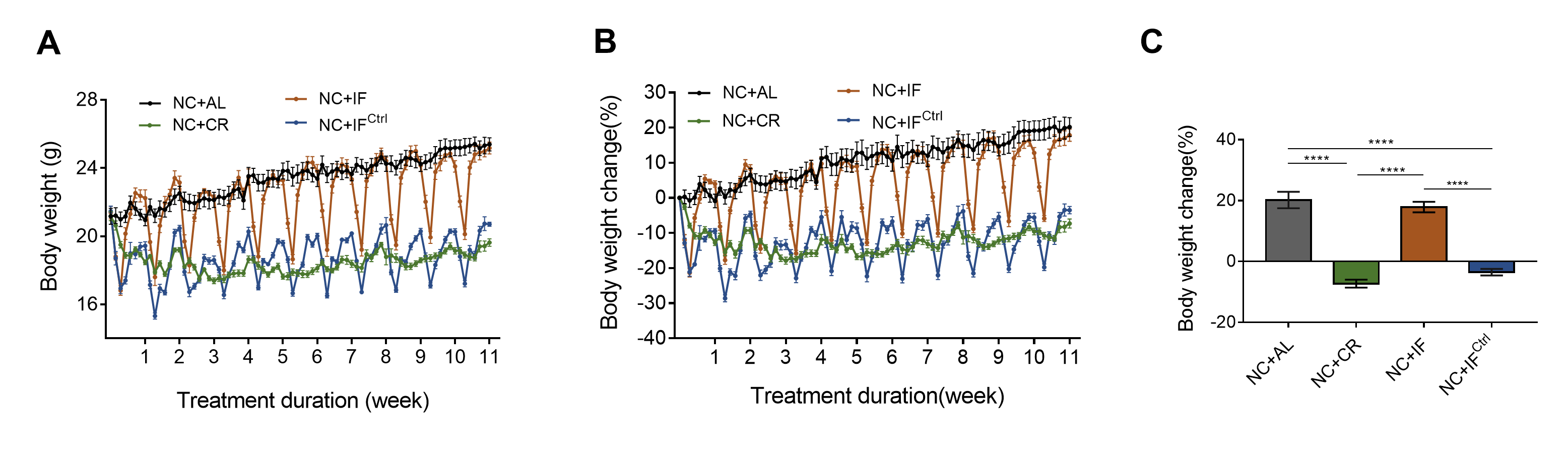

Supplement: Supplementary file 2 — Additional file 2. Body weight of NC-fed mice under different intervention regimens. (A) Body weight curves of NC-fed groups. (B) Body weight change curves of NC-fed groups. (C) Body weight changes of NC-fed groups after 11 weeks of intervention on Day 7 of Week 11. Data are presented as the mean ± S.E.M. For each group, n = 6–7. Data were analyzed using one-way ANOVA followed by Tukey’s post hoc test. *P < 0.05, **P < 0.01, ***P < 0.001, ****P < 0.0001. [file 12915_2021_987_MOESM2_ESM.tif]

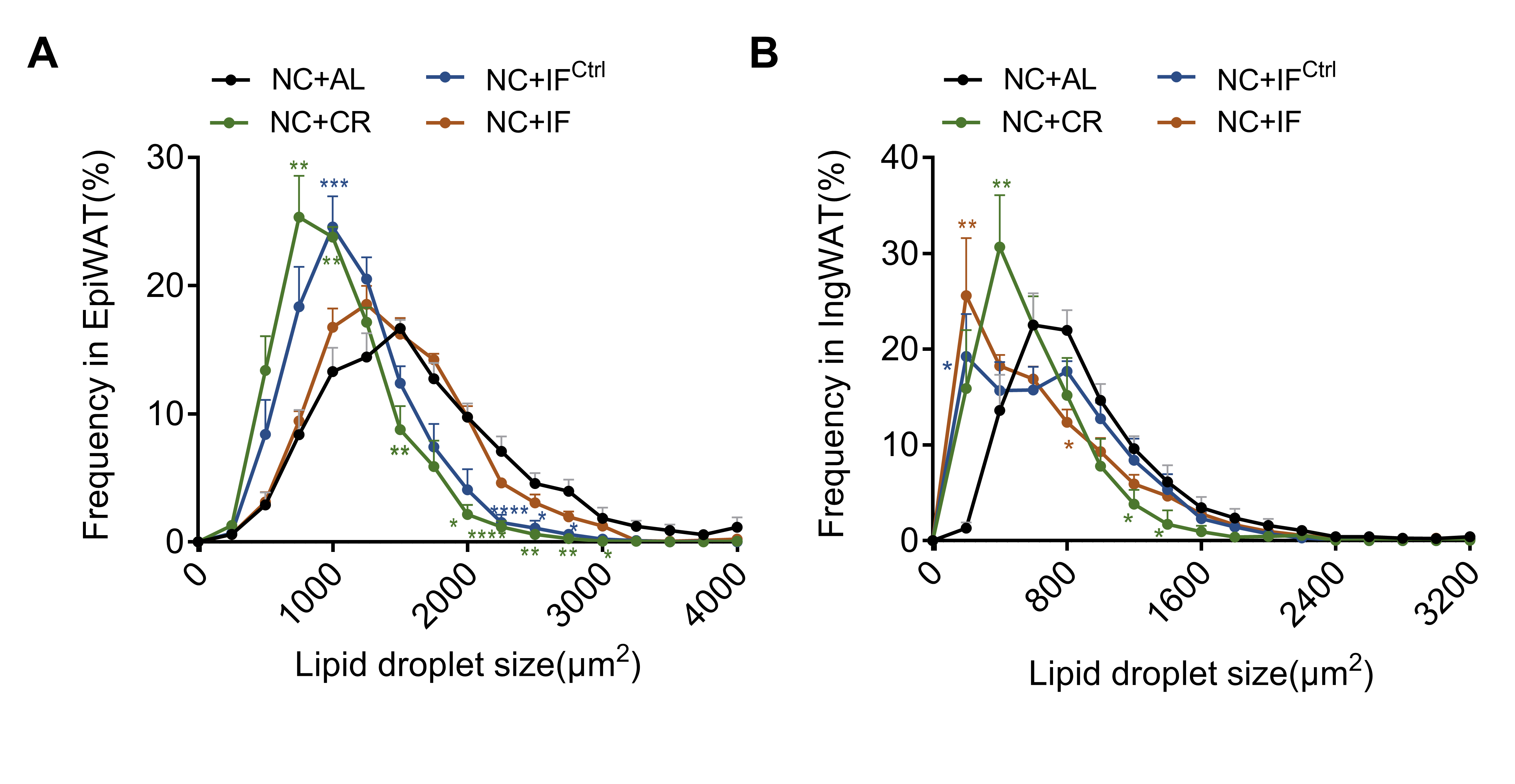

Supplement: Supplementary file 3 — Additional file 3. Lipid droplet size profiling of adipocytes of NC-fed mice under three intervention regimens. Lipid droplet size profiling of adipocytes from (A) EpiWAT and (B) IngWAT of NC-fed mice. Mice were tested after 11 weeks of intervention on Day 7 of Week 11. Data are presented as the mean ± S.E.M. For each group, n = 6–7. Data were analyzed using one-way ANOVA followed by Dunnett’s multiple comparisons to compare groups with the NC + AL group. *P < 0.05, **P < 0.01, ***P < 0.001, ****P < 0.0001. [file 12915_2021_987_MOESM3_ESM.tif]

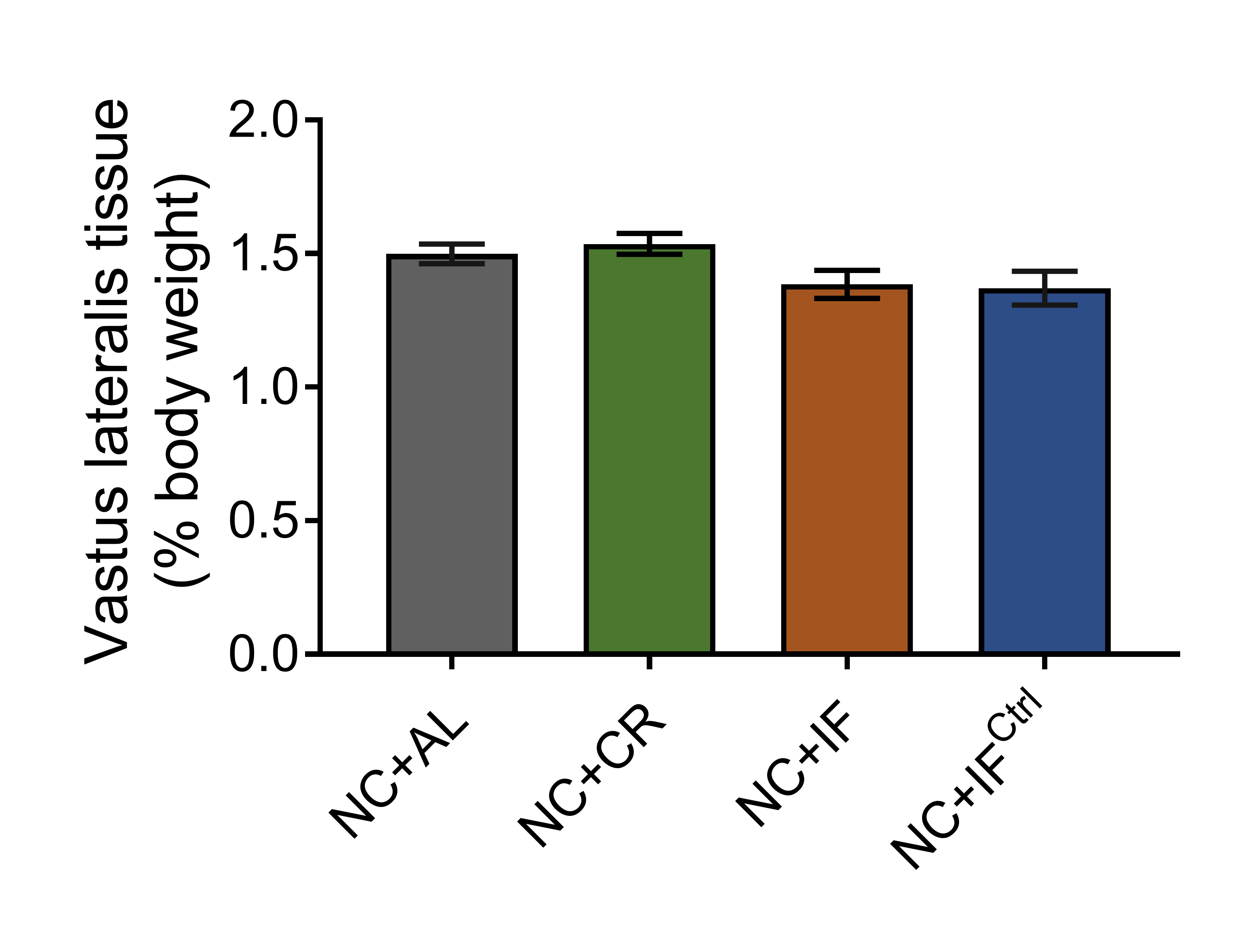

Supplement: Supplementary file 4 — Additional file 4. Vastus lateralis tissue weights as a percentage of body weight of NC-fed groups. Mice were tested after 11 weeks of intervention on Day 7 of Week 11. Data are presented as the mean ± S.E.M. For each group, n = 6–7. Data were analyzed using one-way ANOVA followed by Tukey’s post hoc test. [file 12915_2021_987_MOESM4_ESM.tif]

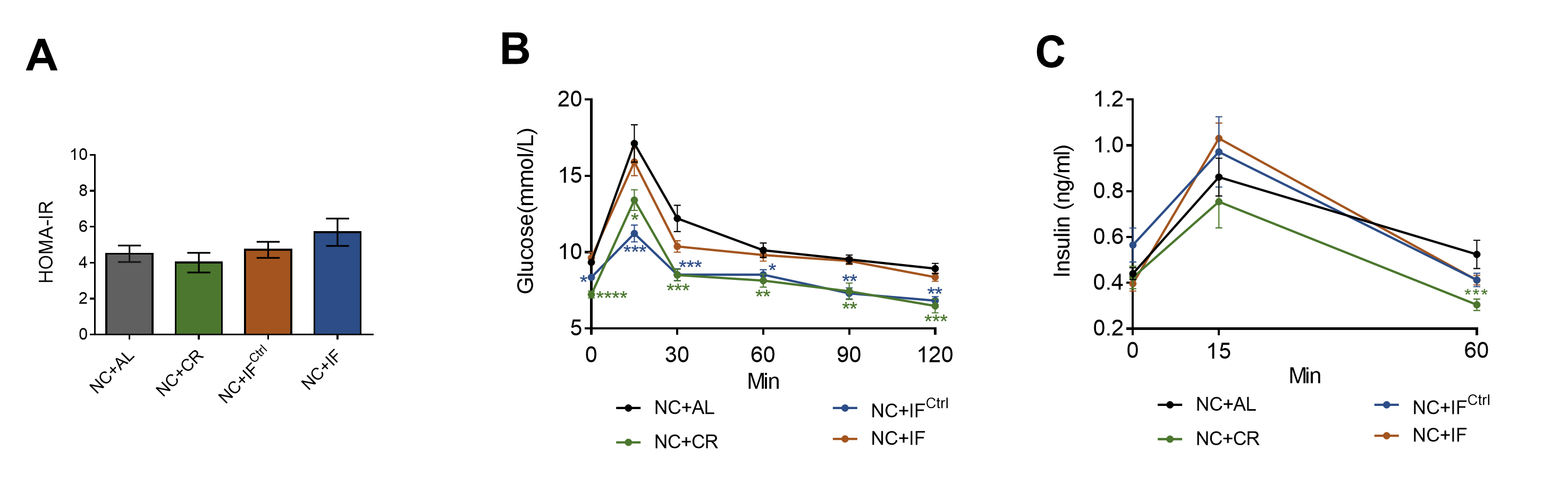

Supplement: Supplementary file 5 — Additional file 5. Glucose metabolism parameters of NC-fed mice. (A) Homeostatic model assessment for insulin resistance (HOMA-IR). (B) Blood glucose curves during the oral glucose tolerance test (OGTT) of NC-fed groups. (C) Serum insulin during the OGTT (0–60 min). Mice were tested after 11 weeks of intervention on Day 7 of Week 11. Data are presented as the mean ± S.E.M. For each group, n = 6–7. Data were analyzed using one-way ANOVA followed by Dunnett’s multiple comparisons to compare with the NC + AL group. *P < 0.05, **P < 0.01, ***P < 0.001, ****P < 0.0001. [file 12915_2021_987_MOESM5_ESM.tif]

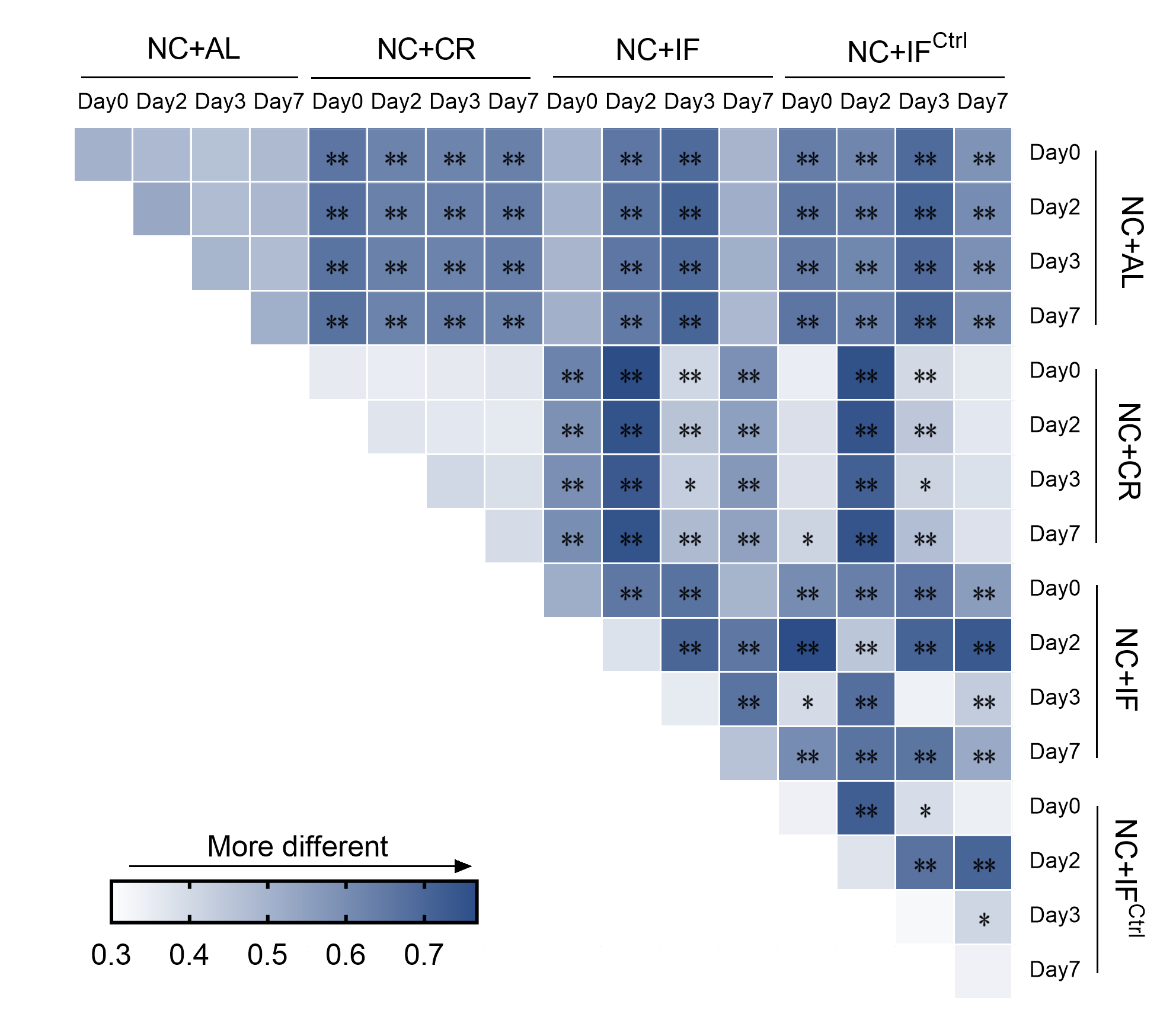

Supplement: Supplementary file 6 — Additional file 6. Bray-Curtis distances of gut microbiota between NC-fed groups at the all time points. Permutational multivariate analysis of variance (PerMANOVA, 9999 permutations) was used to sequentially determine whether the two groups/time points were significantly different. *P < 0.05, **P < 0.01 (with FDR adjustment). [file 12915_2021_987_MOESM6_ESM.tif]

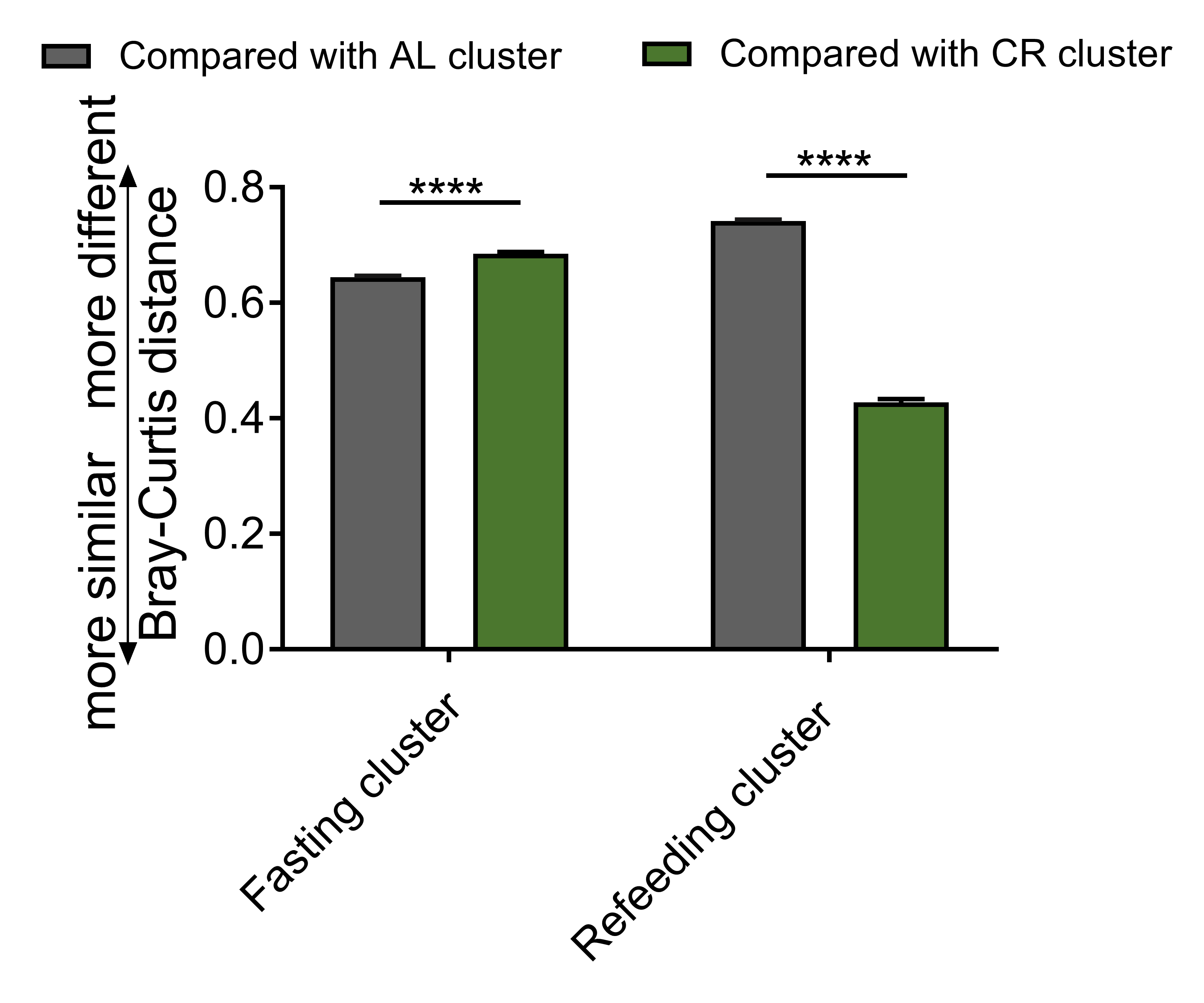

Supplement: Supplementary file 7 — Additional file 7. Intraindividual variations in the gut microbiota of Cluster Fasting and Cluster Refeeding compared with Cluster AL and Cluster CR. Mean values ± SEM are shown. Data were analyzed using the Mann-Whitney U test. *P < 0.05, **P < 0.01, ***P < 0.001, ****P < 0.0001. [file 12915_2021_987_MOESM7_ESM.tif]

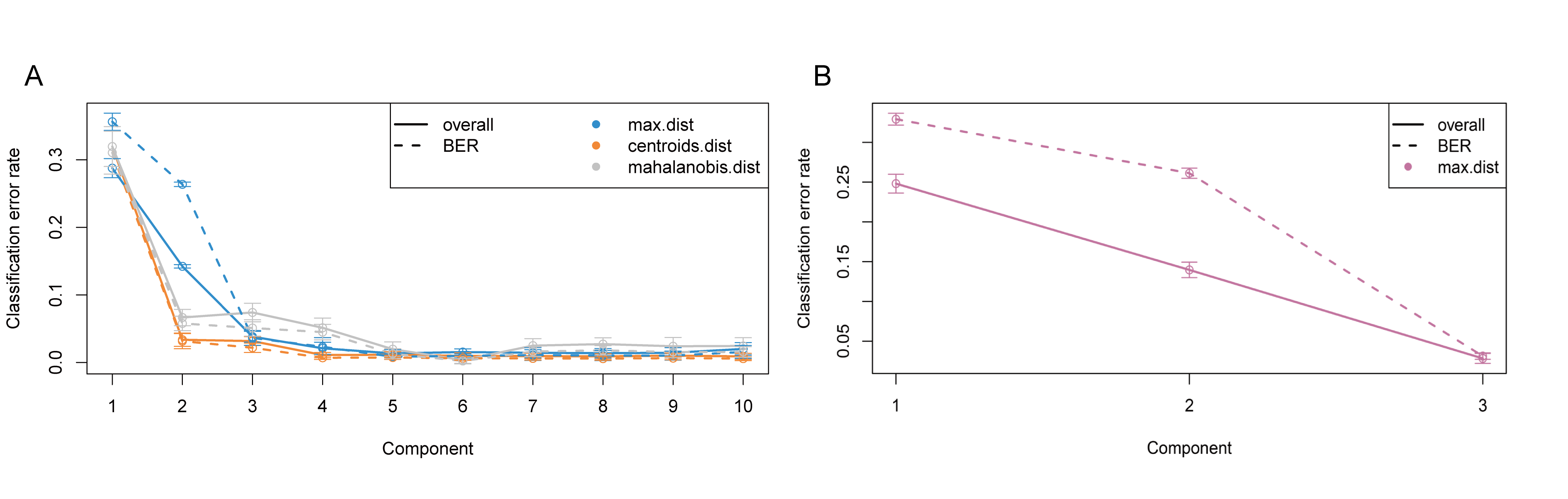

Supplement: Supplementary file 8 — Additional file 8. The overall and balanced error rate (BER) of classification in the sPLS-DA model of NC-fed groups. [file 12915_2021_987_MOESM8_ESM.tif]

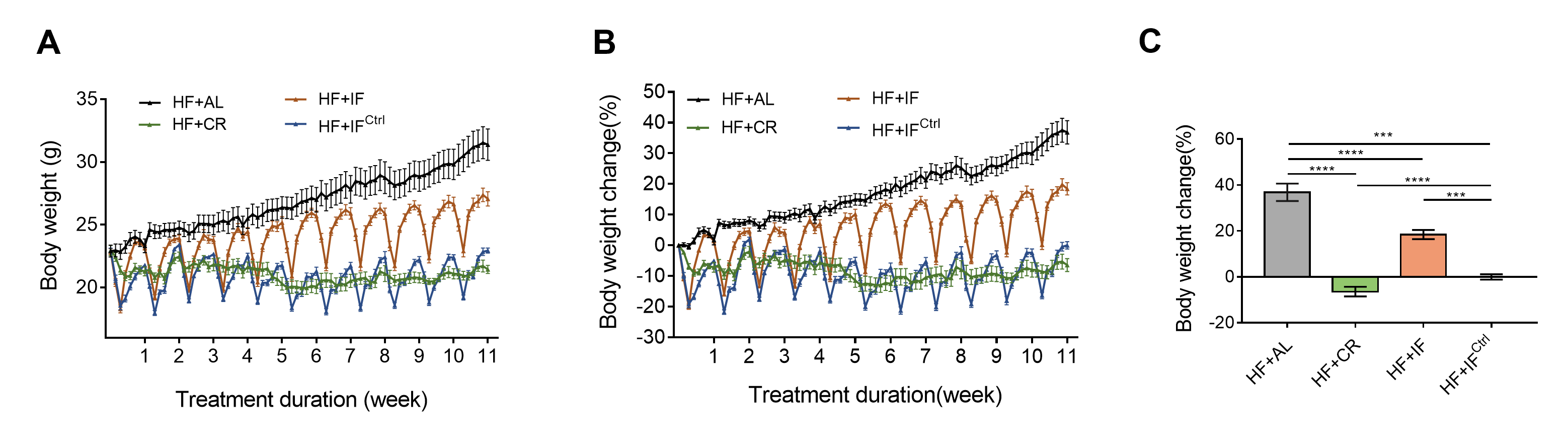

Supplement: Supplementary file 11 — Additional file 11. Body weights in HF-fed mice under different intervention regimens. (A) Body weight curves of HF-fed groups. (B) Body weight change curves of HF-fed groups. (C) Body weight changes of HF-fed groups after 11 weeks of intervention on Day 7 of Week 11. Data are presented as the mean ± S.E.M. For each group, n = 6–7. Data were analyzed using one-way ANOVA followed by Tukey’s post hoc test. *P < 0.05, **P < 0.01, ***P < 0.001, ****P < 0.0001. [file 12915_2021_987_MOESM11_ESM.tif]

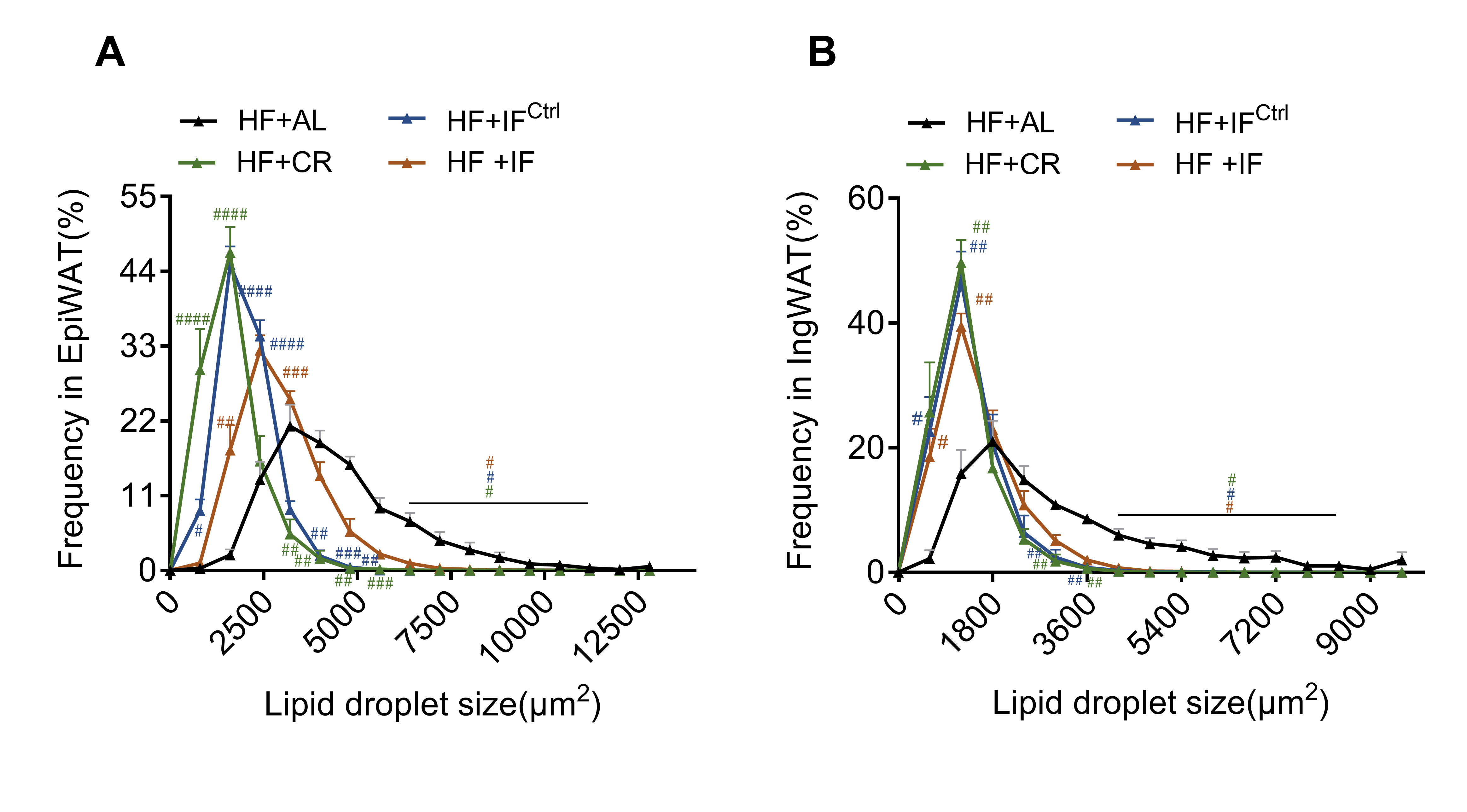

Supplement: Supplementary file 12 — Additional file 12. Lipid droplet size profiling of adipocytes of HF-fed mice under three intervention regimens. Lipid droplet size profiling of adipocytes from (A) EpiWAT and (B) IngWAT of HF-fed mice. Mice were tested after 11 weeks of intervention on Day 7 of Week 11. Data are presented as the mean ± S.E.M. For each group, n = 6–7. Data were analyzed using one-way ANOVA followed by Dunnett’s multiple comparisons to compare with the HF + AL group. *P < 0.05, **P < 0.01, ***P < 0.001, ****P < 0.0001. [file 12915_2021_987_MOESM12_ESM.tif]

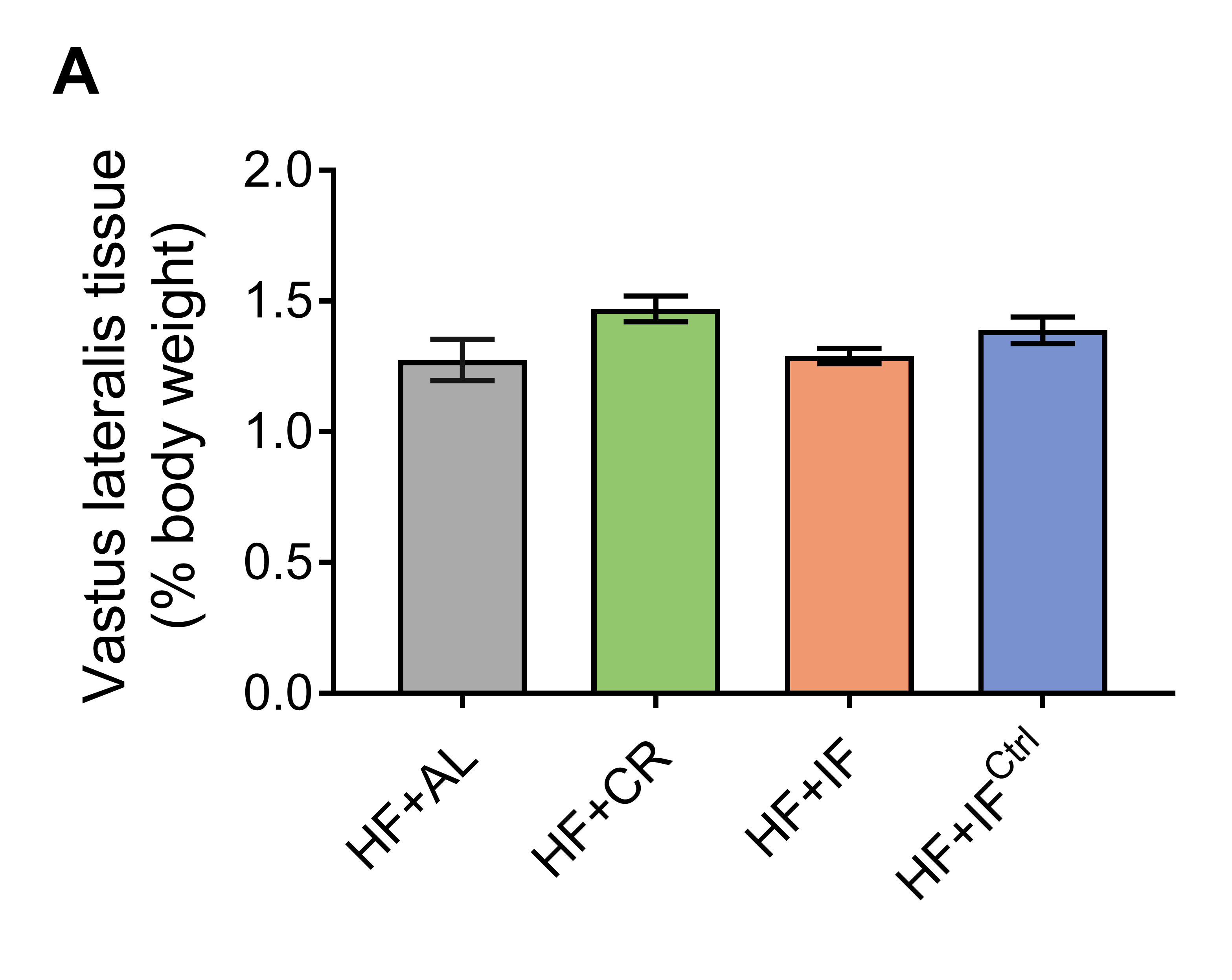

Supplement: Supplementary file 13 — Additional file 13. Vastus lateralis tissue weights as a percentage of body weight of HF-fed groups. Mice were tested after 11 weeks of intervention on Day 7 of Week 11. Data are presented as the mean ± S.E.M. For each group, n = 6–7. Data were analyzed using one-way ANOVA followed by Tukey’s post hoc test. [file 12915_2021_987_MOESM13_ESM.tif]

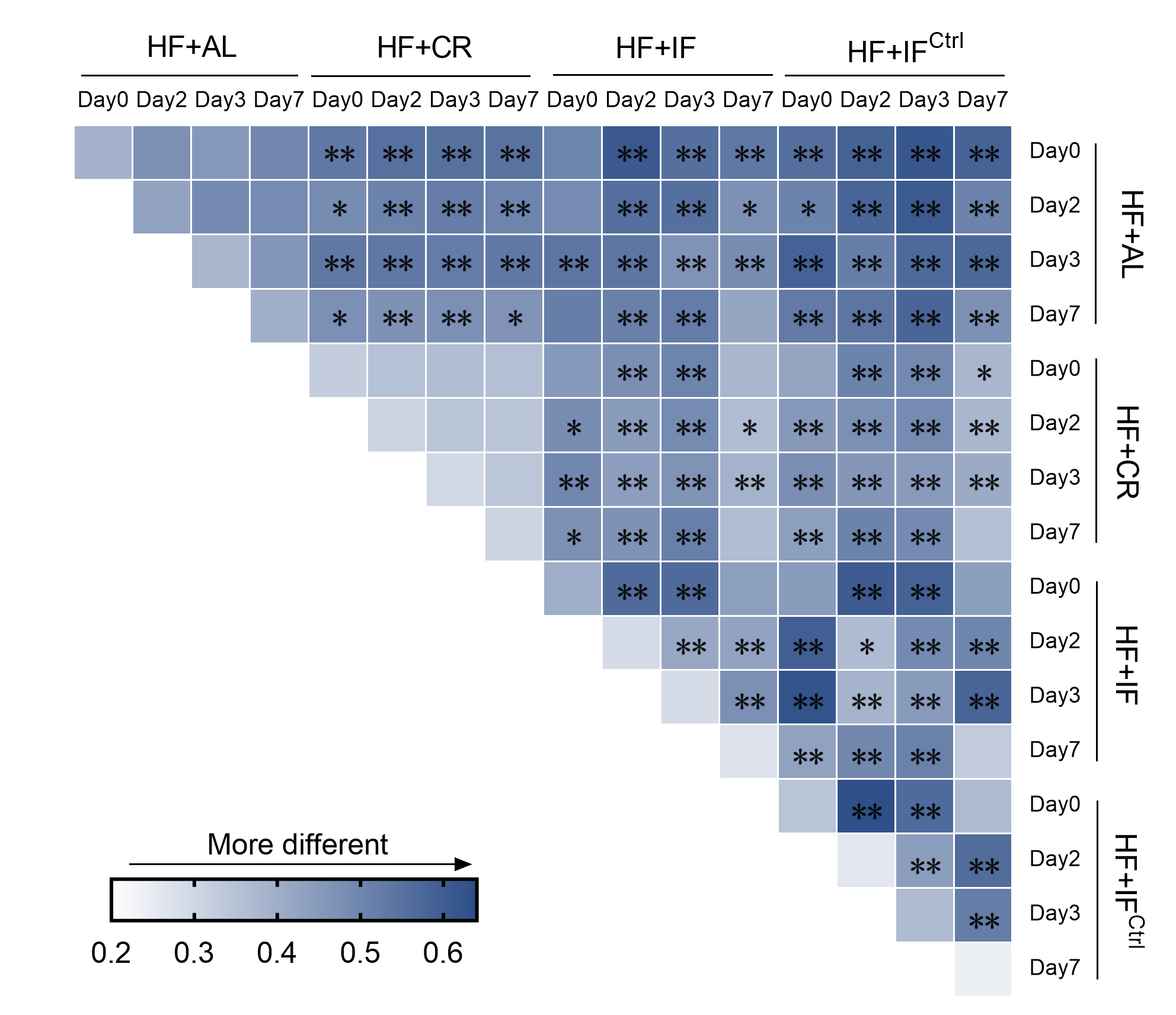

Supplement: Supplementary file 14 — Additional file 14. Bray-Curtis distances of gut microbiota between HF-fed groups at all time points. Permutational multivariate analysis of variance (PerMANOVA, 9999 permutations) was used to sequentially determine whether the two groups/time points were significantly different. *P < 0.05, **P < 0.01 (with FDR adjustment). [file 12915_2021_987_MOESM14_ESM.tif]

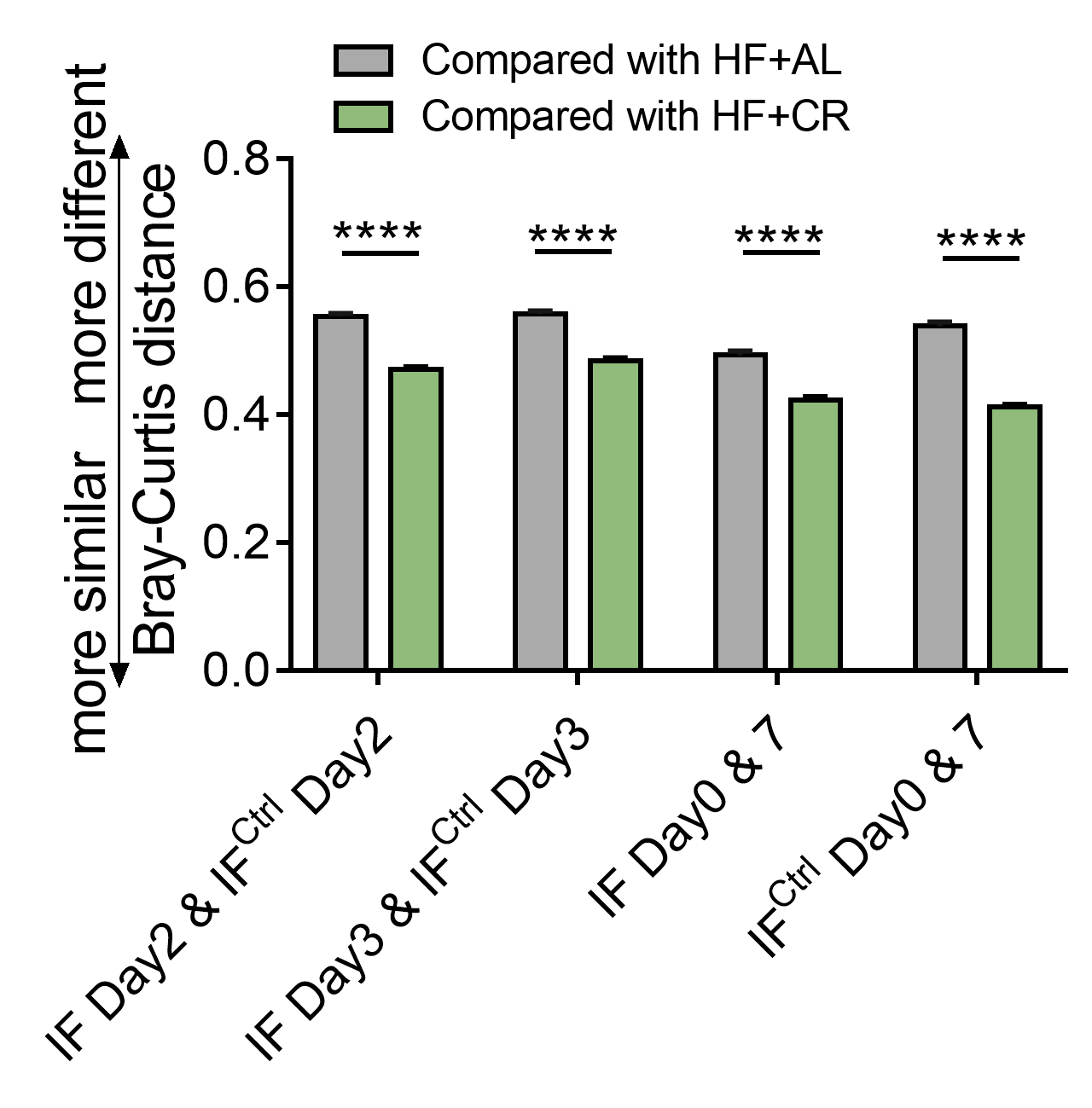

Supplement: Supplementary file 15 — Additional file 15. Intraindividual variations in the gut microbiota of Cluster Day2 (IF Day2/IFCtrl Day2), IF Day3/IFCtrl Day3, IF Day7 and IFCtrl Day7 for the two consecutive weeks compared with Cluster AL and Cluster CR. Mean values ± SEMs are shown. Data were analyzed using the Mann-Whitney U test. *P < 0.05, **P < 0.01, ***P < 0.001, ****P < 0.0001. [file 12915_2021_987_MOESM15_ESM.tif]

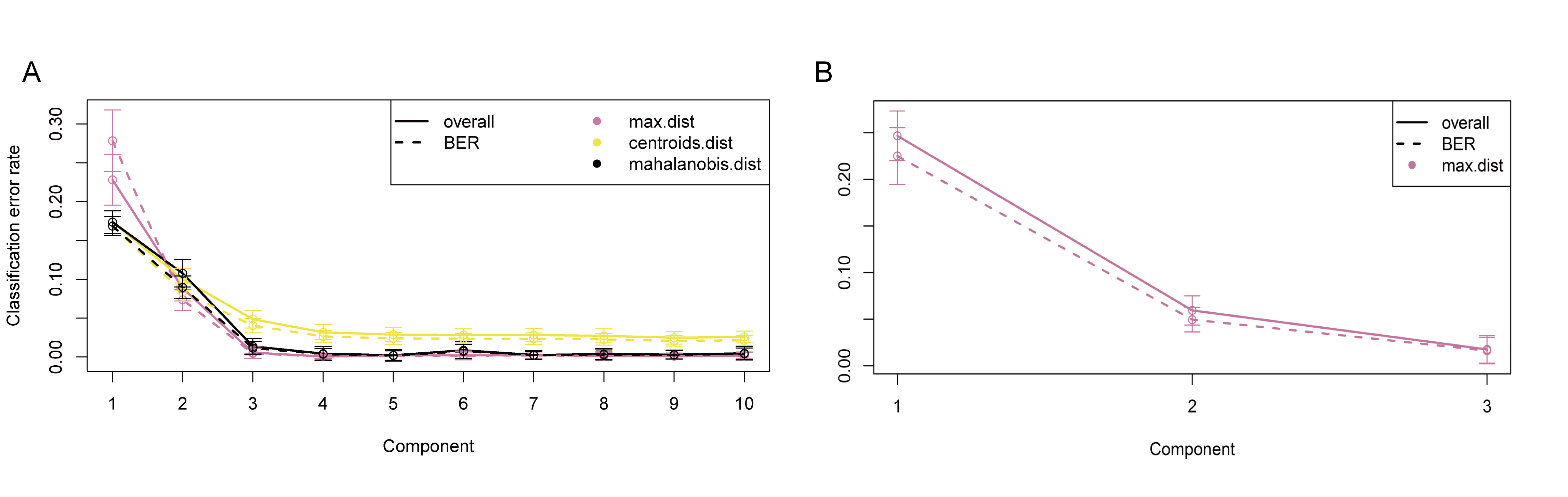

Supplement: Supplementary file 16 — Additional file 16. The overall and balanced error rate (BER) of classification in the sPLS-DA model of HF-fed groups. [file 12915_2021_987_MOESM16_ESM.tif]
